# Comparative Expression Profiles of Midgut Genes in Dengue Virus Refractory and Susceptible *Aedes aegypti* across Critical Period for Virus Infection

**DOI:** 10.1371/journal.pone.0047350

**Published:** 2012-10-15

**Authors:** Chitra Chauhan, Susanta K. Behura, Becky deBruyn, Diane D. Lovin, Brent W. Harker, Consuelo Gomez-Machorro, Akio Mori, Jeanne Romero-Severson, David W. Severson

**Affiliations:** Eck Institute for Global Health, Department of Biological Sciences, University of Notre Dame, Notre Dame, Indiana, United States of America; Johns Hopkins School of Public Health, United States of America

## Abstract

**Background:**

*Aedes aegypti* is the primary mosquito vector for dengue virus (DENV) worldwide. Infectivity of dengue virus varies among natural populations of this mosquito. How *A. aegypti* responds to DENV infection relative to which genes and associated pathways contribute to its differential susceptibility as a vector is not well defined.

**Methods/Principal Findings:**

Here, we used custom cDNA microarrays to identify groups of genes that were differentially expressed in midgut tissues between susceptible and refractory strains in a highly time specific manner. While genes involved in protein processing in the endoplasmic reticulum, mRNA surveillance, and the proteasome were significantly up-regulated in the susceptible strain, several metabolic processes including glycolysis, glycan biosynthesis and Wnt pathway were active in the refractory strain. In addition, several key signaling genes were expressed as common responsive genes in both susceptible and refractory mosquitoes that may be necessary for signal transduction to trigger the appropriate host response to the viral infection. These are coordinately expressed in the form of tight gene networks and expression clusters that may be necessary to differentially contribute to the progression of dengue infection between the two strains.

**Conclusions:**

Our data show that highly correlated differential expression of responsive genes throughout the post infection period in *A. aegypti* midgut tissues is necessary for a coordinated transcriptional response of the mosquito genes to host or defend the viral infection.

## Introduction

Dengue fever (DF) is caused by four antigenically related viruses (DENV1-4) belonging to the family Flaviviridae, genus *Flavivirus*. Disease symptoms present a broad spectrum of illnesses ranging from non-specific febrile illness to the more severe dengue hemorrhagic fever (DHF) and dengue shock syndrome (DSS). Infection by individual serotypes provides long-lasting serotype-specific immunity against reinfection, but only transient heterologous cross-protection to other serotypes [1]–[2]. Instead, secondary infections may increase the likelihood for DHF/DSS [3]–[4]. Over 2.5 billion people are at risk, with ∼50 to 100 million new DF infections each year, including ∼500,000 cases of DHF/DSS resulting in ∼25,000 deaths [5]–[6]. At present, no effective dengue vaccines are available for DF prevention and no specific drugs are available for DF treatment, with treatment therefore limited to palliative care [7]–[8].

DENV transmission is totally dependent on the availability of a mosquito vector competent to transmit virus to a naïve human host ∼7–14 days following blood feeding on a viremic host. *Aedes aegypti* is the primary DENV vector across most tropical and subtropical regions of the world. *A. aegypti* is synanthropic, highly anthropophagic, and readily breeds in natural and artificial containers located peridomestically and even domestically [9]. Historically, DF control has largely been synonymous with *A. aegypti* control, primarily via source reduction of larval habitats and use of larval and adult insecticides. However, for a variety of reasons [10] even intensive mosquito control/eradication programs have not proven sustainable in preventing DENV transmission [11]–[13].

Transmission of DENV through *A. aegypti* requires that the virus successfully infect and replicate in midgut and salivary gland epithelia [14]. Critical stages include invasion of the midgut epithelium, establishment of infection and replication of the virus in the midgut, dissemination of the virus from the midgut to the hemocoel, and viral invasion of and replication in the salivary glands [15]. Known barriers to infection include midgut infection barriers (MIB) targeting early stages of midgut epithelium infection that potentially involve processes such as receptor binding, viral uncoating, transcription or translation, and midgut escape barriers (MEB) involving restricted dissemination of infectious virions to the hemocoel possibly by blocking viral passage throughout the basal lamina or the viral maturation process [15]. The genetic capacity of the mosquito to acquire, maintain, and transmit the virus determines its vector competence. Once DENV escapes from the midgut epithelium to the hemocoel, multiple tissues including the salivary glands become infected [14], [16], [17], and the mosquito is presumably competent to transmit virus [18] if the vector-virus interaction fails to elicit salivary gland infection barriers [15].

Considerable intra-specific variation has been demonstrated in *A. aegypti* vector competence for flaviviruses [15]. While it is well known that insects are capable of mounting an effective innate immune response against invading pathogens [19], [20], the molecular basis for DENV refractoriness in *A. aegypti* has only recently been subject to investigation. Several studies have been performed to define specific gene expression patterns in response to DENV infection; these studies have each used the Rockefeller strain or the derived Rockefeller/UGAL strain [21]–[23], [24]. Both the Toll and JAK-STAT signaling pathways were implicated in suppressing DENV infections in *A. aegypti*. To date, comparisons of gene expression changes between DENV susceptible and refractory stains are limited. This remains a major gap in our knowledge of understanding how gene expression is related to differential vector competence to DENV infection among different mosquito populations. We recently performed a genome-wide transcriptome study of susceptible and refractory *A. aegypti* strains to identify DENV infection responsive genes at two critical early time points post-infection (3 and 18 hr) [25]. We identified a large number of genes (n = 2,504) showing significant differential expression (‘responsive genes’) following viral challenge. The responsive genes were coordinately expressed in distinct modules representing gene networks specific to a susceptible, refractory or common response to infection. In the present study, we report results from a custom cDNA microarray analysis of midgut tissues at several key post-infection time points between a DENV susceptible and a refractory strain of *A. aegypti*. DENV-specific transcriptional responses were identified beginning at one hour post-infection through the critical four day time frame that typically leads to virus escape from the midgut in susceptible individuals. Our results provide additional insights on gene expression patterns specific to susceptible or refractory vector responses to DENV infection and have implications for developing novel control methods.

## Results

### *Aedes aegypti* Genes Show Temporal Expression Patterns Upon DENV Infection

**Figure 1:**
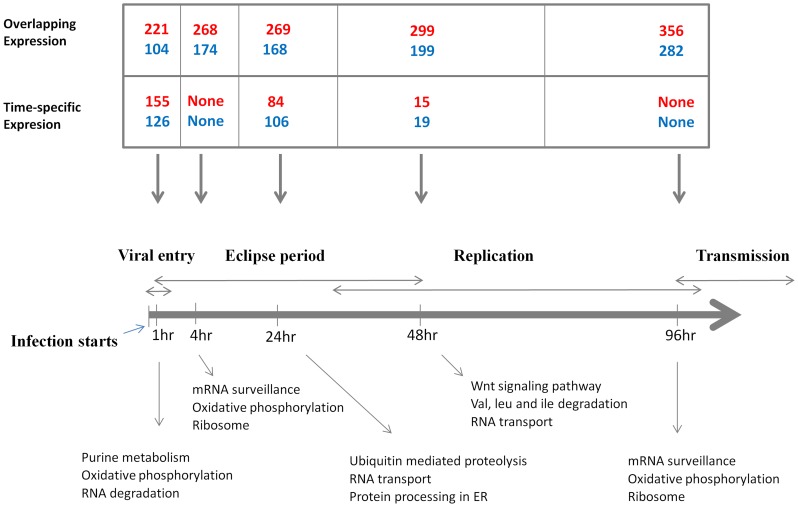
Number and distribution of DETs at different post infection time points. The thick horizontal arrow shows the progression of dengue infection with the time points marked in hours (hr). In the boxes above each time point, the numbers of transcripts that are up-regulated in the susceptible strain or down-regulated in the refractory strain are shown in red. Numbers of transcripts that are up-regulated in the refractory strain or down-regulated in the susceptible strain are shown in blue. The numbers in each box corresponding to ‘Time-specific Expression’ represent DETs which are differentially regulated exclusively at the specified post-infection time. On the other hand, entries in the boxes corresponding to ‘Overlapping Expression’ show numbers of DETs which are differentially expressed at more than one time point after infection (See Table S1). The downward arrows show specificity of expression of the genes to time point(s). The genes showing differential expressions at each time point represent specific pathways genes. The top three representative pathways specific to each infection time point are shown below the time points.

The cDNA microarray experiment was conducted to profile midgut transcript expression between Moyo-S (MS, susceptible to DENV) and Moyo-In-Dry (MD, refractory to DENV) females, upon challenging them with the JAM 1409 strain of DENV (serotype-2). The MD strain was originally collected from Shauri Moyo, near Mombasa, Kenya. MS is a sub-strain of MD. These strains were originally selected for high and low infectivity by *Plasmodium gallinaceum*. Subsequently, these strains have been verified to show significant differences in susceptibility to dengue virus infection. The microarray analysis of 9,504 annotated genes in *A. aegypti* revealed significant differential expression among a number of transcripts at each time point after DENV-2 infection (Table 1). The microarray experiment was conducted using cye3/cye5 dye swap design where the ratio of dye intensity of MS relative to MD was normalized to calculate relative expression changes in the infected and uninfected samples of the two strains. Thus, genes up-regulated in the MS strain are genes that are down-regulated in the MD strain and *vice versa*. The differentially expressed transcripts (DETs) were associated with significant changes in expression (SAM significance threshold delta >0. 29 with average FDR <7%) in the infected samples vs uninfected samples and showed at least 2-fold change in expression levels between the susceptible MS and refractory MD strains. All the significant DETs that are differentially expressed at specific post-infection times or at more than one time point are listed in Table S1. The total numbers of DETs ranged from n = 442 at 4 hr to n = 638 at 96 hr. The detailed analysis of the microarray data indicated that several DETs showed differential expression across multiple and in some cases at all time points, while a relatively small number of DETs showed time point specific expression (Figure 1). We utilized *A. aegypti* pathway predictions available at Kyoto Genes and Genomes (KEGG) in an effort to better define functions of the significant genes. We identified KEGG pathways for 438 genes from our array experiments showing significant differential expression in response to DENV infection. Representative functions of the time specific responsive genes at each point are shown in Figure 1. It should be noted that these are not the only predicted functions, but instead they represent the top three most representative KEGG pathways by the responsive genes at each time point. Of note, the 1 hr post infection time point was where the maximum number of time-specific expression changes occurred (n = 281) compared to any other post-infection times we investigated. At 1 hr post-infection, a total of 155 transcripts showed >2-fold higher expression in the susceptible strain than the refractory strain. Another suite of transcripts (n = 126) were responsive at this time point but showed >2-fold higher expression in the refractory strain compared to the susceptible strain. These transient expressions at 1 hr post-infection were associated with specific biochemical pathways such as nucleotide metabolism, energy metabolism and RNA degradation, among others. Of note, the 4 hr and 96 hr time points were different, wherein no 4 hr or 96 hr time-specific gene expression was observed and instead these genes were responsive at other post infection times including a subset of genes responsive at 1 hr post-infection. A common set of genes associated with the energy metabolism process (particularly, oxidative phosphorylation) was identified at 1 hr, 4 hr and 96 hr post-infection indicating that host energy machinery is significantly affected at these infection times as a part of the mosquito response to the pathogen. Finally, we observed that the top three KEGG pathways represented across all post-infection time point were mainly associated with RNA related activities, proteolysis, protein synthesis (mRNA surveillance, ribosome) as well as signal transduction.

**Table 1:** Number of differentially expressed genes at each post-infection time point.

Post-infection Time point	Susceptible_up/Refractory_down	Refractory_up/Susceptible_down
1 hr	336	230
4 hr	268	174
24 hr	353	274
48 hr	314	218
96 hr	356	282

The 2^nd^ column shows the total number of significant genes which are up-regulated in the Moyo-S strain but down-regulated in the Moyo-D strain at the corresponding time point. The 3^rd^ column shows the total number of significant genes which are up-regulated in the Moyo-R strain but down-regulated in the Moyo-S strain at the corresponding time point.

The significant genes were also annotated based on predicted gene ontologies of *A. aegypti* genes available at VectorBase (http://www.vectorbase.org). Several gene ontologies including immune response, RNAi interference, programmed cell death, translation and protein folding and mRNA metabolism and processing were identified that each constituted 1–6% of the significant genes (Figure S1). About 51% of the significant genes could not be annotated to gene ontologies as many of these are hypothetical or unknown genes.

### Susceptible Versus Refractory Transcriptional Response

**Figure 2:**
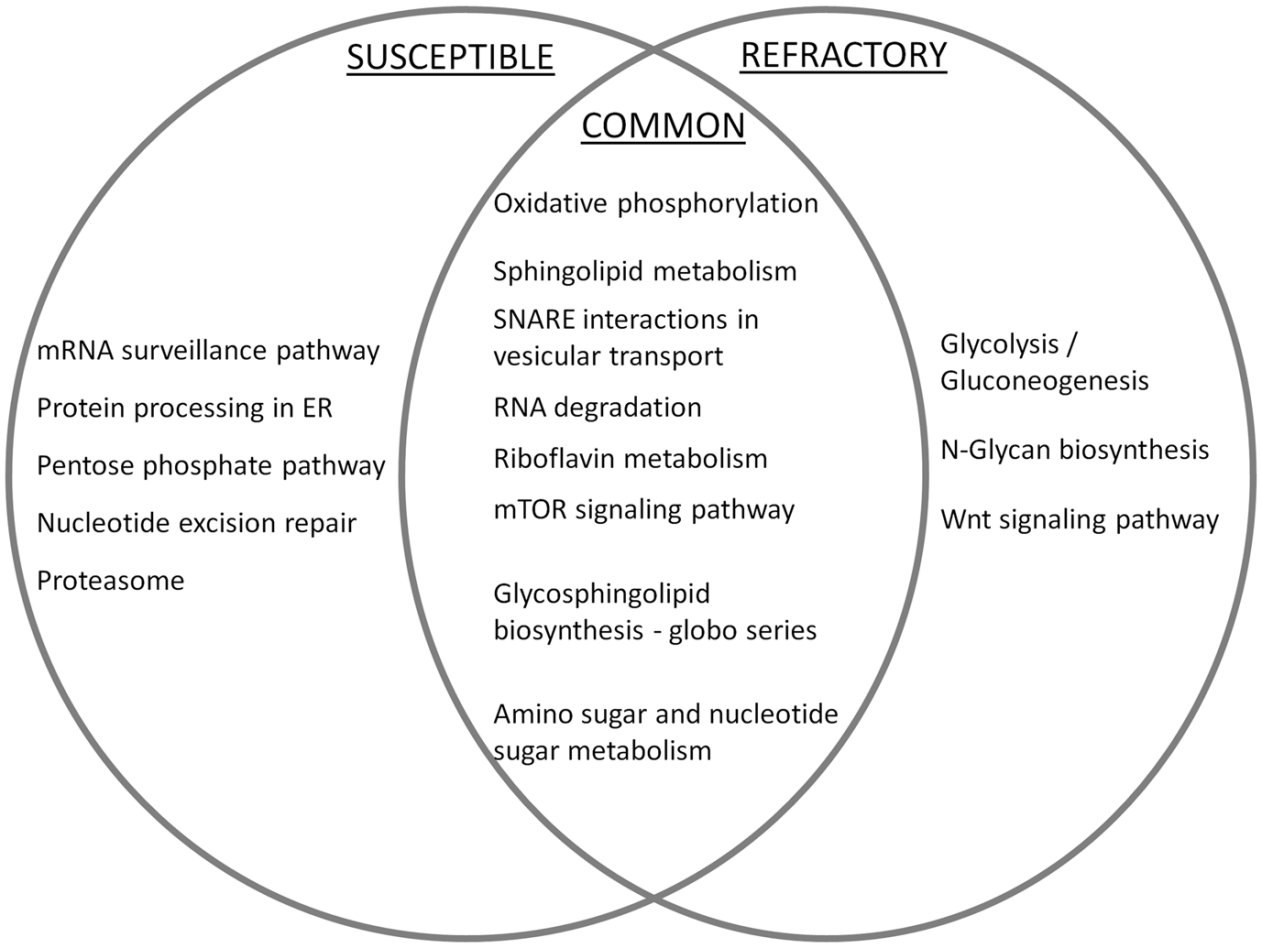
Differential distribution of KEGG pathway genes in susceptible versus refractory strains. Pathways where the number of significant genes are significantly higher in susceptible than refractory strain are shown specific to ‘susceptible’ and pathways where the significant genes that show the opposite distribution pattern are shown specific to ‘refractory’. Several pathways were found commonly activated in both the strains without statistically significant bias and they are listed as ‘common’ pathways.

By calculating the statistically significant differences in the number of genes up-regulated in the susceptible and down-regulated in the refractory strain and *vice versa* between post-infection time points (1 hr vs. 4 hr, 4 hr vs. 24 hr, 24 hr vs. 48 hr and 48 hr vs. 96 hr), we found eight specific KEGG pathways that were significantly different (Fisher’s exact test, p<0.05) between the susceptible and the refractory strain gene responses. Of these, five pathways were significantly biased for up-regulated genes in the susceptible strain and three pathways were biased for up-regulated genes in the refractory strain (Figure 2). Genes related to mRNA surveillance, protein processing in endoplasmic reticulum, proteasome, nucleotide excision repair and pentose phosphate pathways were up-regulated in the susceptible strain whereas genes related to specific metabolic pathways (glycolysis and glycan biosynthesis) were significantly up-regulated in the refractory strain. The susceptible-specific pathways were largely related to a single KEGG module (genetic information processing pathways) in contrast to refractory-specific pathways which were mostly related to metabolism processes. Apart from susceptible- and refractory-specific pathways, the results showed eight pathways that were up-regulated in both the strains without any bias. Nearly equal numbers of genes belonging to each of these pathways were differentially expressed in both the strains suggesting that these pathways play a common role in both the strains upon dengue virus infection.

### Correlated Expression Patterns of Responsive Genes

**Figure 3:**
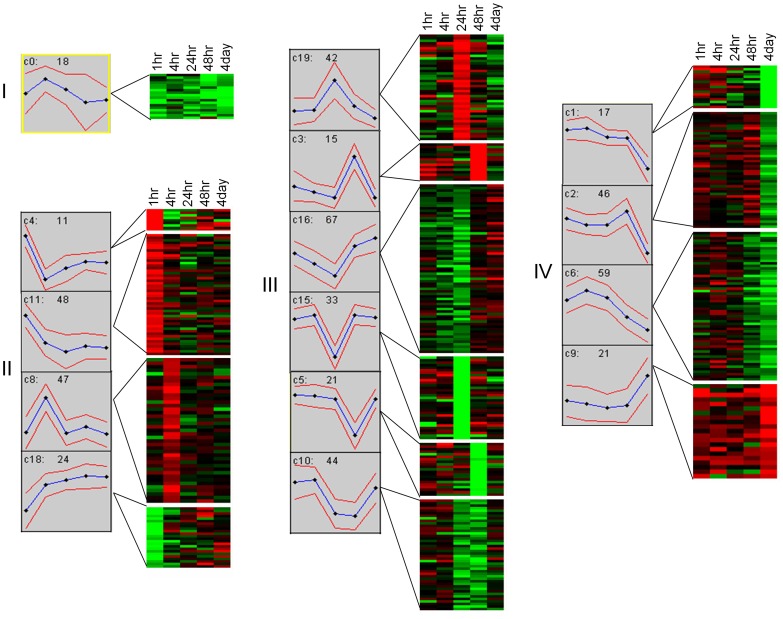
Cluster analysis and self organizing maps of DETs. Four groups (I–IV) are shown that represent similar patterns of gene expression. For each group, the expression patterns of DETs (Moyo-D relative to Moyo-S) are shown at the 5 time points. In each graph, the y-axis represents the k-means of expression of transcripts and x-axis represents the post-infection time points. The blue line shows the mean expression values of the transcripts (number of genes showing that pattern is listed on the top of each graph) and the two red lines above and below the blue line shows the level of variance of transcript expression at each time point. The c# is the cluster number. The higher mean values indicate the genes are expressed at significantly higher levels in Moyo-D than in Moyo-S. The self organizing maps are shown to the right of the corresponding cluster graphs. Red and green colors represent up-regulation and down-regulation of genes in Moyo-D relative to Moyo-S, respectively.

We identified 20 clusters (cluster# 0–19) of differential transcript expression among the DENV responsive genes using the GeneCluster program. These showed highly correlated expression throughout the five post infection time points as assessed by the k-means clustering method. These individual clusters were further grouped into four major groups by constructing self organizing maps (SOMs) of the responsive transcripts (Figure 3). In each group, the responsive transcripts showed similar patterns of up- and down-regulation of expression at each post-infection time point. The annotated gene lists of the differentially expressed transcripts are included in Table S2.

#### Group I

The genes in group I (cluster: c0) were down-regulated in MD and/or up-regulated in MS at all the five time points. One of the significant genes identified in group I was *importin β1* (AAEL006667). This gene encodes a nuclear import receptor that targets proteins to the nucleus. It is consistently unregulated in the MS but not in the MD strain suggesting a role in the progression of infection in susceptible midguts.

#### Group II

In this group, gene clusters showed higher expression at the 1 hr or 4 hr time points than other time points. We observed a larger number of genes and clusters showing higher expression in MD and/or lower expression in MS (c4, c8, c11) as compared to the opposite (c18). One of the genes identified in this group was AAEL011453, coding for a putative galactose specific C-type lectin. The over-expression of this C-type lectin in the MD strain as early as 4 hr post DENV infection could imply a possible role of the gene product in restricting infection in a refractory population.

#### Group III

The gene clusters in group III showed higher expression at 24 hr and/or 48 hr time points. In this group, a large number of genes were up-regulated in the MS strain but down-regulated in the MD strain (c5, c10, c15, c16) at these two points. Conversely, 57 genes (c3, c19) were up-regulated in the MD strain but down-regulated in the MS strain. In group III, we observed several genes that are known to play a role in host-virus interactions. One of these, AAEL006271, encodes superoxide dismutase (SOD) which showed higher expression in our DENV susceptible strain. We found that another gene, AAEL006794, with a putative role in host defense against viral infection had higher levels of expression in our susceptible strain. This gene codes for dicer-2, a component of the RNA interference (RNAi) pathway. We also observed at least five proteases in this group, viz. AAEL007432 (putative serine collagenase precursor), AAEL006425 (trypsin), AAEL009722 (putative clip-domain serine protease), AAEL013907 (d-alanyl-d-alanine carboxypeptidase) and AAEL004793 (dipeptidyl peptidase).

#### Group IV

Gene clusters with their peak expression at 4 days post-infection were grouped as IV (c1, c2, c6, c9). In this group, the majority of the genes were up-regulated in the MS strain and down-regulated in the refractory strain MD (c1, c2 and c6). Only ∼15% genes of this group showed the opposite pattern of expression in that they were up-regulated in the MD strain but down-regulated in the MS strain (c9). In group IV, we identified genes involved in different cellular process such as endocytosis (AAEL008374) and regulation of autophagy (AAEL010516) which are up-regulated in the susceptible strain but down-regulated in the refractory strain indicating a possible role of the genes in viral entry into the epithelium cells and processes required for virus maturation and dissemination from the midgut. The same expression group also included the Hedgehog signaling pathway gene (AAEL004351) that is activated only in the refractory strain. Other genes that are activated along with this signaling gene included genes required for ribosome biogenesis, as well as glycerophospholipid metabolism (AAEL008865, AAEL008865, AAEL005583). Additionally genes involved in phosphatidylinositol signaling system (AAEL012326), ubiquitin mediated proteolysis (AAEL008374) and proteasome activity (AAEL006061) were also differentially expressed in group IV implying extensive functions in cellular and signaling processes in response to dengue infection.

**Figure 4:**
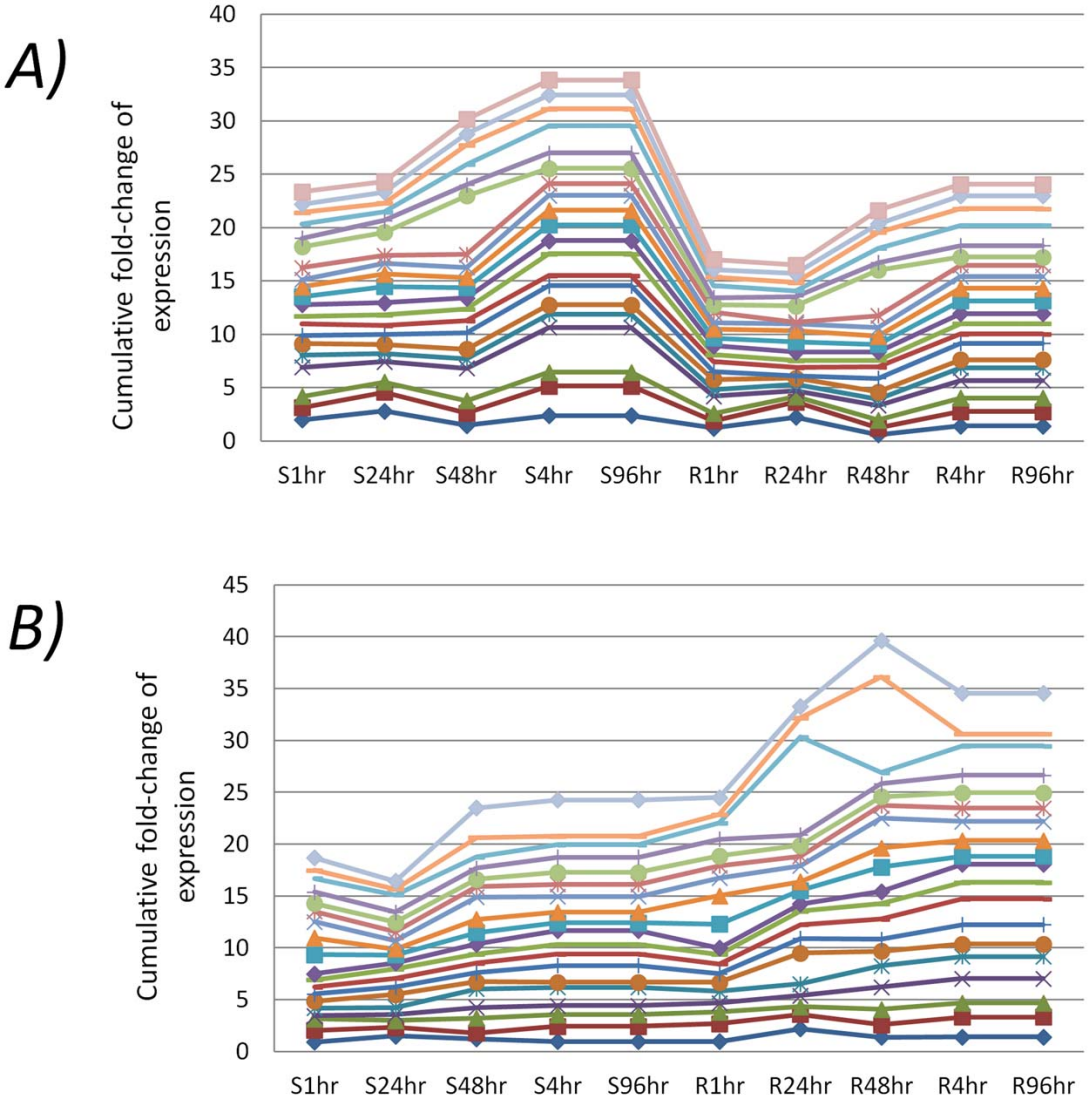
Examples of highly correlated gene expression throughout post-infection times. (A) Transcripts that had higher levels of expression in the DENV susceptible strain than the DENV refractory strain and (B) Transcripts that had higher levels of expression in the refractory strain than the susceptible strain. The expression levels are shown as stacked line graphs where the x-axis shows strains and time points and y-axis shows the cumulative values of fold-changes in expression of the responsive genes.

**Figure 5:**
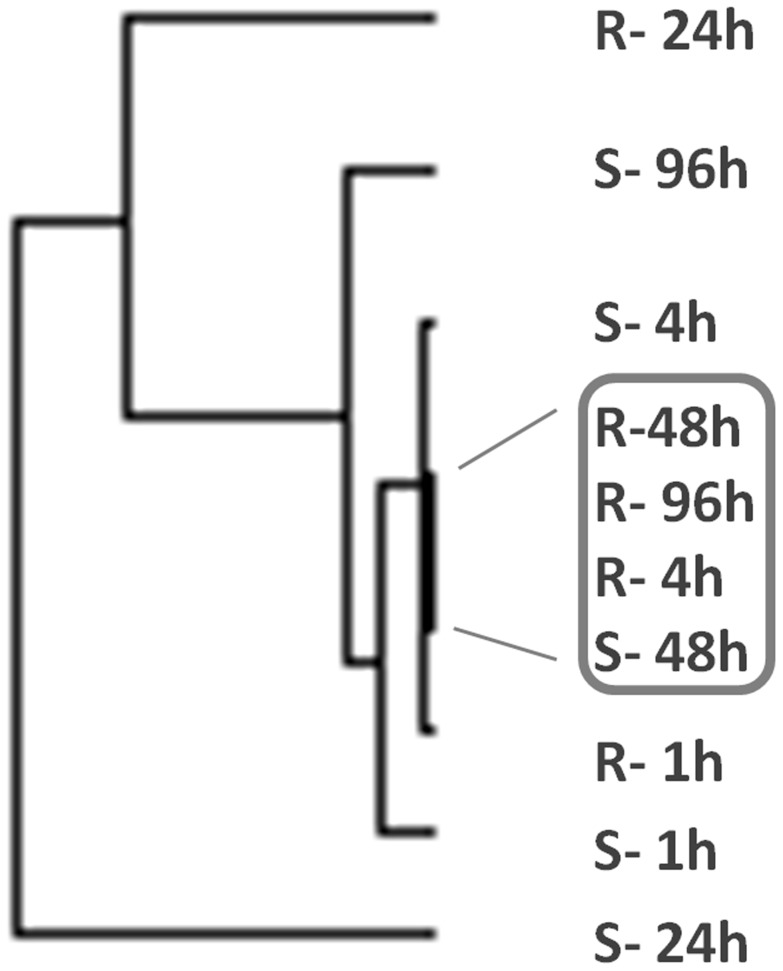
Hierarchical cluster of transcript expression among DENV susceptible (S) and DENV refractory (R) strains based on differential expression among post infection time points after DENV infection. The time points and strains within the box show higher correlated expression after DENV infection than other time points.

The highly correlated (Pearson correlation >0.8) expressed genes between the susceptible and refractory strains are listed in Table S3. They have higher levels of expression in the susceptible strain than the refractory strain (n = 20) where the expression changes were correlated throughout the post-infection times (Figure 4A). Another set of genes (n = 19) was also found that showed similar correlations but in the opposite direction, wherein they showed higher expression in the refractory strain than the susceptible strain (Figure 4B). These genes, when mapped to the k-means clusters, corresponded to cluster#0 and cluster#9 (Figure 3). Based on variation of gene expression between MS and MD mosquitoes at the five post-infection time points, the hierarchical cluster analysis showed that gene expression tends to cluster at specific times after infection. The cluster tree of gene expression levels among the ten branches (2 strains×5 time points) clearly suggests that gene expression changes in MS at 48-hr and those in MD at 4-hr, 48-hr and 96-hr are highly similar (Figure 5). Because both the strains show highly similar expression changes at the same post-infection time point (i,e, 48-hr), the 48-hr post infection time point may be critical for DENV infection of both the strains (Figure 5). The genes differentially expressed in MS at 48 hr also show high correlation with MD genes differentially expressed at 4 hr and 96 hr. At 24 hr, the susceptible and the refractory mosquitoes show the least correlation in gene expression suggesting that this might be the ‘defining moment’ of vector competence between the two strains to DENV infection. These expression patterns clearly suggest highly temporal modulation of gene expression in *A. aegypti* to invoke susceptible and refractory interaction with DENV.

We also discovered significant genes that are associated with different signal transduction pathways including MAPK, JAK-STAT, Wnt signaling, mTOR signaling, phosphatidylinositol signaling and hedgehog signaling systems, some of which were expressed with higher fold-changes in the refractory strain than the susceptible strain and *vice versa* (Table 2). In addition, genes related to endocytosis, peroxisome and ribosome synthesis were also identified that are differentially expressed between the two strains (Table 3). It is interesting to note that genes associated with endocytosis and peroxisomes define similar cellular processes critical for transport and catabolism functions in the cell. Thus, we suspect that these genes might be involved in gene networks for triggering the appropriate host response (susceptible or refractory) to DENV infection. Moreover, these genes show strong network associations among each other suggesting extensive cross-talk among them (Figure S2). It is thus likely that these genes play significant roles in the cross-talk among the responsive genes to modulate mosquito-virus interactions during these early post-infection periods.

**Table 2:** List of signal transduction genes that show differential expression (up-regulation in one and down-regulation in the other) between MS and MD strains in response to DENV infection.

cDNA ID	Gene ID	Gene name	Time	Fold change (susceptible)	Fold change (refractory)	Pathway
NACNJ53	AAEL008234	dishevelled	24-hr	10.074	0.457	Wnt signaling pathway
NABVF55	AAEL007035	tak1	1-hr	1.176	2.521	Wnt signaling pathway
NAAFJ23	AAEL003539	WD-repeat protein	48-hr	0.452	1.655	Wnt signaling pathway
NABVF55	AAEL007035	tak1	48-hr	1.090	7.049	Wnt signaling pathway
NADAL87	AAEL012326	calmodulin	48-hr	1.817	0.869	Phosphatidylinositol signaling
NABTF26	AAEL008179	RAS homologueenriched in brain(RHEB)	1-hr	3.071	1.208	mTOR signaling pathway
NABTF26	AAEL008179	RAS homologueenriched in brain(RHEB)	24-hr	1.000	2.457	mTOR signaling pathway
NADBK82	AAEL010488	ets	1-hr	0.893	5.112	MAPK signaling pathway
NADVW55	AAEL004319	epidermal growth factor receptor	1-hr	0.862	2.524	MAPK signaling pathway
NADBK82	AAEL010488	ets	24-hr	0.460	2.031	MAPK signaling pathway
NABXP83	AAEL006949	suppressor of cytokine signaling	1-hr	2.076	0.555	Jak-STAT signaling pathway
NABRY87	AAEL010619	costal 2	24-hr	1.091	0.523	Hedgehog signaling pathway

The fold-change values of expression are shown for each transcript and time of observation.

**Table 3:** List of significant genes identified from the microarray experiment which are related to endocytosis, peroxisome and ribosome functions.

cDNA ID	Gene ID		Gene_name	Time	Fold-change (susceptible)	Fold-change (refractory)
**Endocytosis**						
**NAAGR39**	AAEL001014		vacuolar protein sorting-associated	1-hr	3.161	0.711
**NADW531**	AAEL005271		Rab11 family-interacting protein	1-hr	5.590	0.476
**NAAG590**	AAEL006370		amsh	24-hr	1.098	0.525
**NABU821**	AAEL012219		ubiquitin specific protease	24-hr	2.122	0.848
**NABYA27**	AAEL006460		par-6 gamma	4-hr	6.814	1.122
**NABYA27**	AAEL006460		par-6 gamma	96-hr	6.814	1.122
**NAAG590**	AAEL006370		amsh	1-hr	0.445	0.902
**NABWA74**	AAEL005339		hepatocyte growth factor-regulated tyrosine kinase substrate (hgs)	1-hr	0.728	3.598
**NADZW22**	AAEL010834		arf GTPase-activating protein	4-hr	1.174	2.982
**NADZW22**	AAEL010834		arf GTPase-activating protein	96-hr	1.174	2.982
**Peroxisome**						
**NAAF684**	AAEL001601		protein Mpv17	48-hr	2.307	0.765
**NAAF684**	AAEL001601		protein Mpv17	4-hr	1.284	0.578
**NABXE43**	AAEL009112		Mpv17-like protein	4-hr	1.736	0.638
**NAAF684**	AAEL001601		protein Mpv17	96-hr	1.284	0.578
**NABXE43**	AAEL009112		Mpv17-like protein	96-hr	1.736	0.638
**Ribosome**						
**NACAK05**	AAEL003572		RNAse h (70)	48-hr	2.058	0.590
**NABWH80**	AAEL004526		U3 small nucleolar RNA-associated protein 13	1-hr	0.888	4.244
**NACAK05**	AAEL003572		RNAse h (70)	1-hr	1.234	3.023
**NACAK05**	AAEL003572		RNAse h (70)	4-hr	1.004	2.087
**NACAK05**	AAEL003572		RNAse h (70)	96-hr	1.004	2.087
**NABY626**	AAEL004175		40S ribosomal protein S17	1-hr	1.493	0.225
**NADDG77**	AAEL011656		40S ribosomal protein S15	24-hr	1.392	0.684
**NABRS19**	AAEL005901		40S ribosomal protein S3a	4-hr	1.749	9.186
**NADZ349**	AAEL005097		cold induced protein (BnC24A), putative	4-hr	1.029	7.400
**NABRS19**	AAEL005901		40S ribosomal protein S3a	96-hr	1.749	9.186
**NADZ349**	AAEL005097		cold induced protein (BnC24A), putative	96-hr	1.029	7.400

The time points at which the gene (gene ID, the gene name and the cDNA shown) are significantly differentially expressed between the two strains are shown. The fold changes of expression with respect to the control are shown in both the strains. The genes are activated in antagonistic manner between the two strains suggesting possible role of coordinated action of these three groups of genes in the mosquito after dengue virus infection.

### Validation of Microarray Expression Data

We validated the microarray expression data by qRT-PCR of eight randomly selected genes. The relative expression levels of each of the selected genes for both mosquito strains at three randomly chosen time points was compared to the uninfected controls (see Methods for details). Comparison of log_2_ ratios of MD/MS expression data of qRT-PCR results with the microarray expression data showed similar patterns in each case (Figure S3). The correlation coefficient (r^2^) was greater than 0.7 between qRT-PCR and microarray data among the eight transcripts at each time point.

### Signature of Flavivirus Induced Transcriptome

Our results further suggest that many of the differentially expressed genes identified from the current investigation are common genes that are induced in *A. aegypti* by DENV and other flaviviruses. The 4 hr post infection time point from the present study was the only close time point that could be compared with the 3 hr post infection time point of the previous study by Behura et al. 2011 [25]. From the current study, we identified a total of 442 ESTs (of which 358 have been annotated for known genes) which are differentially expressed in midguts between MS and MD at the 4 hr post infection time. From the Behura *et al*. 2011 [25] study, a total of 594 genes were identified that were differentially expressed in whole carcass between MS and the refractory Moyo-R (MR) strain (DENV infection rate ∼20%). By comparing these significant genes between the two studies, we identified a total 207 genes that were up-regulated at 3 hr post infection in MS in the Behura *et al*. 2011 experiment and a very similar number (n = 203) genes that were up-regulated in MS in the current experiment. A total of 118 genes were common between the two gene sets (Table S4). These data indicate that in spite of 1 hr time differences in the infection status, and differences in experimental designs (midgut tissues in the present study and whole carcasses in the previous study), nearly 50% of the genes show common patterns of expression between susceptible and refractory strains across the two studies. We further extended the comparison with another study by Colpitts *et al*. 2011 [24]. The microarray study by Colpitts *et al*. 2011 was conducted to identify *A. aegypti* responsive genes upon infection with yellow fever virus, dengue virus and West Nile virus. As two post-infection time points (1 day and 2 day were common between that study and our present study), we compared the responsive genes between studies. We found that despite differences in the putative susceptible mosquito strains used in these two studies (Rockefeller strain in Colpitts *et al.* 2011 vs. MS in the present study), a total of 219 genes were identified as significantly expressed by both studies (Table S5). Most of these genes were differentially expressed after 1 day of infection in both experiments. At 2 day post-infection time, only a few genes showed differential expression in both. At least 21 genes (shown in bold in Table S5) showed similar fold-changes in expression at the same post-infection time in both studies. A total of 100 transcripts showed significant differential expression, but in opposite directions (upregulated in MS strain but downregulated in Rockefeller strain and *vice versa*) across the two studies. Many of these common genes identified from both the studies are associated with gene ontology terms such as intracellular activity, protein binding functions, zinc ion binding, catalytic activity and nucleic acid binding proteins (data not shown). Importantly, three genes (AAEL000832, AAEL007854 and AAEL013068) which are related to mRNA surveillance and found significant in our study were also significant in the Rockefeller strain at the same post infection times. This suggests that a common core set of genes may have functional roles in DENV susceptibility although the genetic background of the mosquito strains may also have an influential role in modulating the expression of vector genes.

## Discussion

We performed a time course gene expression profiling of midgut tissues from DENV refractory and susceptible strains of *A. aegypti* in response to infection with a high passage DENV-2 strain from Jamaica (JAM 1409). Successful transmission of dengue virus by *A. aegypti* requires a successful series of biological events following acquisition of an infectious blood meal. These events include invasion of the midgut epithelium via endocytosis, establishment of infection and replication of virus in the midgut, dissemination of the virus from the midgut to the hemocoel and viral invasion of and replication in the salivary glands. The two early time points (1 hr and 4 hr) were selected to target differentially expressed genes involved in early events associated with virus entry into the midgut epithelial cells. After successful entry into the epithelial cells, the virus undergoes transcription and translation followed by assembly of infectious virus particles. These are released from the cells and the infection spreads to the nearby cells. In a study of DENV tropism and kinetics of infection in orally infected *A. aegypti* strains, DENV infected epithelial cells were observed as early as 48 hr post-infection [26]. Thus, the middle time points (24 hr and 48 hr) were likely to capture the genes involved in the replication and assembly process as well as early release and infection of nearby cells. We expected that by 4 days post-infection, DENV dissemination from the midgut epithelium would likely have occurred in a susceptible mosquito host. Thus, the 4 day time point was chosen to target the genes involved relatively late in life cycle events of the virus such as maturation and dissemination processes.

The different groups of gene expression clusters seem to have important roles in the differential host response of *A. aegypti* to dengue infection during different post infection times. The *importin β1* (AAEL006667) gene identified in the group I cluster encodes a nuclear import receptor that targets proteins to the nucleus. The DENV NS5 protein, which is an RNA dependent RNA polymerase, has a high affinity nuclear localization signal (NLS) for importin β [27]. The high sequence conservation of the importin β binding NLS among NS5 proteins from dengue and related flaviviruses implies an important role in flavivirus infectious cycles [27]. Thus, its persistent upregulated expression in the susceptible MS strain may have implications for the higher infectivity of these mosquitoes to DENV. The gene AAEL011453, identified in group II cluster, codes for a putative galactose specific C-type lectin that could impact vector competence in *A. aegypti* owing to its potential role in innate immunity. In *D. melanogaster*, a galactose specific C-type lectin has been shown to participate in the immune response via a hemocyte mediated mechanism [28]. The lectins have a carbohydrate recognition domain that recognizes the glycosylated envelope protein of influenza virus and elicits the innate immune response [29]. This gene is upregulated in the refractory MD strain as early as 4 hr post DENV infection suggesting that it may have a role in restricting infection in this mosquito population. The upregulated expression of AAEL006271, coding for superoxide dismutase (SOD), in group III cluster could be the result of oxidative stress caused by higher levels of DENV infection in these mosquitoes as it has been shown that DENV infection causes oxidative stress in adult human dengue patients [30]. In these patients, SOD was shown to have significantly higher activity as compared to healthy controls. We also identified a gene for dicer-2 (AAEL006794) that is activated in the susceptible MD strain, and is known to play a role in the RNA interference (RNAi) pathway [31]. The RNAi pathway functions as a common antiviral mechanism in mosquitoes and *Drosophila* [31]–[34]. The formation of partially double stranded intermediates during DENV replication can induce the RNAi pathway [31]. Dicer-2 participates in the first step of the RNAi pathway by recognizing and cleaving the double stranded RNA molecules into 20–22 nt fragments leading to complete degradation of the target RNA. Identification of significant expression of several proteases in the group III cluster suggests their role in mosquito vector competence to DENV infection. Some previous studies have reported the insect immune response includes induction of proteases [35]–[37]. In particular, midgut trypsins and serine proteases have been shown to influence the rate of dengue infection and dissemination in *A. aegypti* [38], [39]. In group IV cluster, we identified genes involved in different cellular process such as endocytosis as well as genes for cellular and signaling processes suggesting activation of these pathways in *A. aegypti* in response to dengue infection. In spite of these interesting observations, we think that further studies are required to evaluate the functional significance of the identified genes. For example, it will be interesting to determine if importin β differential expression is required for interaction with the DENV NS5 product in mosquito cells. Most of the existing information on this aspect is obtained from studies on mammalian cells.

In a recent study [40], we found that transcriptional responses of the MS and MR strain genes are significantly influenced by specific intrinsic features of the genes such as codon bias, intron content, gene neighborhood and presence or absence of orthologous and/or paralogous copies in the *A. aegypti* genome. Because we have used progenitor MD strain as the refractory strain in the current study, we wanted to know if the responsive genes of this strain also show similar features in gene structures. Based on preliminary analysis, without running the regression models described in [40], we found that genes which are intronless, low-biased in codon usages but high in gene context (with neighboring genes within 1 kb) and are derived in the *A. aegypti* genome (no or few orthologs in other mosquito species) are relatively more representative in the responsive genes than genes that remain statistically insignificant in expression changes after DENV infection (data not shown). This suggests that MD and derived substrains may be influenced by such gene features in responding to DENV infection. It remains to be investigated if this association is also evident in other *A. aegypti* populations in response to DENV infection.

In summary, the current work shows extensive gene cluster patterns and gene networks that likely reflect the appropriate midgut phenotypic response (susceptible or refractory) to DENV infection. We have also observed similar results in whole carcasses from our earlier work [25]. It was however expected that some differences would be observed between the two studies as the strains, tissues and time points of investigation are different. Moreover, the current study utilized a custom cDNA microarray whereas our earlier work was a genome-wide oligonucleotide microarray. In spite of these differences, we found evidence from both the studies that highly correlated differential expression of responsive genes throughout the post infection times is reflected in a coordinated transcriptional response of the mosquito to host or defend a DENV infection. Moreover, a number of genes identified from this study have also been recognized to have a significant role in common flaviviral infections in *A. aegypti* (24). This suggests that although DENV/*A. aegypti* interaction is modulated by both the viral and mosquito genotypes [41], a core suite of genes may play a significant role in vector competence to DENV infection across the diversity of genotypes represented in mosquito populations.

## Materials and Methods

### Ethics Statement

This study was performed in accordance with the recommendations in the Guide for the Care and Use of Laboratory Animals of the National Institutes of Health. The animal use protocol was approved by the University of Notre Dame Institutional Animal Care and Use Committee (Study # 11–036).

### Mosquito Rearing

The MOYO-S (MS: susceptible to DENV) and MOYO-In-Dry (MD: refractory to DENV) strains of *A. aegypti* were used. The average infection rate of MS is ∼54%, whereas that of MD is ∼13% [42]. Strain origins and history were previously described [43]. Mosquitoes were reared and maintained in an environmental chamber following our standard conditions [44].

### DENV Cell Culture

Cell culture and mosquito infections were performed as previously described [42]. DENV serotype 2 JAM1409 strain was used to infect mosquitoes. The virus was propagated in C6/36 *A. albopictus* cells. Cells were grown in 75 cm^3^ flasks at 28°C in MEM-EBSS media (HyClone SH3002401) with 0.025 mg/ml Gentamycin, 1×(0.01 mM) non-essential amino acids and 10% fetal bovine serum. When ∼80% confluent, the cells were infected with DENV-2 at 0.1 multiplicity of infection (MOI) and incubated in maintenance media supplemented with 2% FBS for 7 days.

### Oral DENV Infections

Equal parts of infectious cell suspension and warmed defibrinated sheep blood (Colorado Serum Company, Denver, CO, USA) were fed to the mosquitoes as an infectious blood meal. For the uninfected control mosquitoes, uninfected C6/36 cell suspension was mixed with sheep blood. Our standard artificial blood feeding protocol using sausage casing as the covering for the artificial membrane feeder was followed [41]. Females were allowed to feed for ∼10 min. Fully engorged females were maintained at 28°C and ∼80% RH and provided with 5% sucrose solution *ad libitum*. Batches of infected and uninfected MS and MD females were removed 1 hr, 4 hr, 24 hr, 48 hr and 4 days post blood meal (PBM) and immediately frozen at −80°C until midguts were dissected. Four independent infectious blood feeding experiments were performed.

### RNA Isolation and Sample Preparation

About 10 midguts from the DENV2 infected females per time point were pooled and RNA was isolated using TRIzol reagent (Invitrogen, Carlsbad, CA, USA) according to the manufacturer’s protocol. The frozen mosquitoes were placed on ice chilled petri dishes while dissecting the midguts to minimize RNA degradation. RNA isolated from a pool of ∼30 midguts each from uninfected blood fed MS and MD females for each time point was used as the reference sample. The extracted RNA quality was assessed by Bioanalyzer as per manufacturer’s instruction.

Following extraction, the RNA was treated with 1.0 U DNase I (Invitrogen, Carlsbad, CA, USA) according to manufacturer’s instructions. First strand cDNA synthesis and labeling was performed using 500 ng of total RNA using the Genisphere 3DNA® Array 900 kit (Genisphere, Hatfield, PA, USA) for each dye, cyanine 3 (Cye3) and cyanine 5 (Cye5), according to the manufacturer’s protocol.

### Microarray Content and Design

Custom microarrays were generated from cDNA amplicons. The cDNAs were constructed from a variety of tissue sources during an effort to generate a large collection of ESTs for assisting in *A. aegypti* genome annotation [45]. Consensus EST assemblies were generated at the *A. aegypti* Gene Index (http://compbio.dfci.harvard.edu/tgi/cgi-bin/tgi/gimain.pl?gudb=a_aegypti). Clones for the array were selected to represent putative unique gene sequences from the tentative consensus (TC) sequence assemblies, with a preference for clones with insert sizes ranging from 500–800 bp. Selected cDNA clones were manually re-plated into 99×96-well microplates.

Clone inserts were PCR amplified using plasmid vector specific primers and bacterial culture templates. The amplicons were purified on a Beckman Coulter Biomek FX robot using Montage PCR 96 Cleanup kits (Millipore, Billerica, MA, USA). The purified amplicons were re-suspended in 100 µl of water, evaporated overnight, and the pellets were re-suspended in 30 µl of 3X SSC spotting buffer. A total of 9,504 cDNA amplicons and 96 blanks were spotted in triplicate onto CMT-Gaps II (Corning, Corning, NY, USA) slides using the Genomic Solutions OmniGrid 100 arrayer at 22°C and 45% RH. Spotted slides were post-processed by baking at 80°C for three hr, incubation in 1% SDS for 2 min, 2 min incubation in 95°C water, plunged 20 times into 100% ethanol at −20°C, and air-dried via centrifugation at 1000 RPM for 5 min.

### Microarray Hybridization

Each hybridization experiment compared the reference RNA to that of DENV-2 infected MS or MD mosquitoes for each of the five time points PBM. Hybridization experiments were carried out following the two step protocol as recommended by the manufacturer (Genisphere, Hatfield, PA, USA). All of the hybridization comparisons included one dye-swap in order to eliminate dye fluorescence bias. Microarray studies included a total of four biological replicate samples. After hybridization and washing, the microarray slides were scanned at two wavelengths, 532 and 635 nm, using the GenePix Pro 4200A scanner (Molecular Devices Corp., Sunnyvale, CA, USA).

### Data Analysis

The hybridization intensities of individual spots were quantified using the segmentation and data analysis software GenePix Pro 6.0 (Molecular Devices Corp., Sunnyvale, CA, USA). The microarray data has been submitted to ArrayExpress under the accession number E-MTAB-46 (http://www.ebi.ac.uk/arrayexpress/experiments/E-MTAB-46). The average signal intensities were normalized with an intensity dependent (Lowess) normalization using GeneSpring GX 7.3 software (Agilent, Santa Clara, CA, USA). The Significance Analysis of Microarrays (SAM) version 2.1 program [46] was used to identify genes with significant changes in gene expression. For the current study, we used 7% as the average false discovery rate (FDR) to minimize the chances of missing important genes.

The genetic networks of differentially expressed genes were constructed by using the ‘GeneNet’ package implemented in *R* [47]. The program uses graphical Gaussian models (GGMs) to represent multivariate dependencies of genes based on high-dimensional (such as microarrays: multi sample+multi time points) expression data. The algorithm estimates the partial correlation (pcor) matrix that is then used to measure shrinkage estimators of covariance of gene expressions [48]. Though graphical Gaussian models are generally applied to independent and identically distributed data, GeneNet incorporates provisions for small scale datasets where the observation time points are unequally spaced. We used the expression data at the five time points of significant genes up-regulated in the susceptible strain and refractory strain to separately estimate dynamic partial correlations. We also performed the network analysis of genes that showed correlated expression patterns between the two strains. The input data for this estimation was generated by use of the ‘longitudinal’ program included in the GeneNet package as a dependency. Once the shrinkage estimator of pcor values are generated, the program performs GGM selection by multiple testing of false discovery rates and that is then used to define the nodes and edges of the association network by an empirical Bayes approach [49]. The graphical view of the networks was either created by using Graphviz 2.18 (http://www.graphviz.org/) or the pair-wise pcor values (that joins one to another) were extracted for further analyses.

GeneCluster version 2.1.7 [50] was used for expression cluster analysis and for generating self organizing maps (SOM) of genes significantly differentially expressed among all the five post infection time points. For each self organizing map, the relative expression values of the genes were reproduced as varying intensities of green and red colors using the TreeView program (version 1.60) (http://rana.lbl.gov/EisenSoftware.htm). The clusters of expression variation of the genes among the five time points were assessed by Euclidean k-means method implemented in GeneCluster. A hierarchical clustering of gene expression was also performed to determine clusters of gene expression based on post-infection time points (*i.e.* which time points show similar expression changes).

The gene annotations of the cDNAs were performed by reciprocal blast against the gene build AaegL1.1 of *A. aegypti* (http://www.vectorbase.org). In order to understand the functional characteristics of the DENV responsive genes, we determined if specific pathways were over-represented. The *A. aegypti* pathway genes were obtained from KEGG (Kyoto Encyclopedia of Genes and Genomes; http://www.genome.jp/kegg-bin/show_organism?org=aag). The signal transduction genes were identified based on gene annotation of KEGG pathways of ‘Signal Transduction’. The number of significant genes associated with different pathways and the total number of *A. aegypti* genes annotated by KEGG for the corresponding pathways are listed in Table S6. They were used along with the total number of KEGG genes identified as significant and the total number of KEGG genes represented by entire microarray to calculate if the significant genes are significantly biased to specific pathway(s). We assumed a null hypothesis where the annotated KEGG pathways had a non-biased representation relative to the observed distribution of DENV responsive genes between mosquito strains and infection time points. Fisher’s exact test was used to determine significance of bias in the distributions of responsive genes to individual pathways (significance threshold was p = 0.05).

### Quantitative Real-time PCR Analysis

Expression levels of selected genes were measured by quantitative real-time PCR (qRT-PCR) analysis using SYBR Green dye technology (Applied Biosystems, Foster City, CA, USA). Primer Express Software version 3.0 (Applied Biosystems) was used to design primers (Table S7). All amplifications and fluorescence quantification were performed using an ABI 7500 Fast System Sequence Detector System (Applied Biosystems) and the Sequence Detector Software version 1.3 (Applied Biosystems). The reactions were performed in a total volume of 25 µL containing 12.5 µL of SYBR Green PCR Master Mix, 300 nmol of each primer, and nuclease free water. Reactions were performed with the following conditions: 50°C for 2 min, 95°C for 10 min followed by 40 cycles of denaturation at 95°C for 15 s, annealing and extension at 60°C for 1 min. Expression values were obtained by using the delta-delta cycle threshold (ΔΔC_T_) method [51] using the ribosomal protein S17 (*RpS17*) gene as the reference control [52]. Log_2_ of the MD/MS gene ratios were compared with the observed microarray expression profiles.

## Supporting Information

Figure S1 **Functional annotations of dengue virus responsive genes.** The percentages of responsive genes (in both the strains at all the five time points) are shown for different gene ontology (GO) terms. The GO terms are listed with color codes corresponding to the chart.
(TIF)

Figure S2 **Gene networks of responsive genes that showed highly correlated expression patterns throughout the five post infection time points between susceptible and refractory strains.** The nodes of the networks (in circles) represent different genes and the connecting lines represent the interaction patterns. Thickness of individual lines represents either strong or weak interactions determined by partial correlation of expression levels by GenNet software. The network shown here represents only a portion of the entire network.
(TIF)

Figure S3 **Comparison of microarray expression data with qRT-PCR data of eight transcripts at three time points chosen randomly (A: 1-hr, B: 24-hr and C: 48-hr).** The X-axis represents qRT-PCR data and Y-axis represents the array data. The linear line shows the correlated expression pattern (r^2^>0.7) in each case.
(TIF)

Table S1 **List of differentially expressed transcripts (DETs) at different post-infection time points.** The DETs in red are up-regulated in Moyo-S but down-regulated in Moyo-D. The DETs in blue are up-regulated in Moyo-D but down-regulated in Moyo-S. The post infection time(s) at which the transcripts are differentially expressed are shown in bold.
(XLSX)

Table S2 **Gene annotations of DETs.** Clusters of transcript expression were identified by using the GeneCluster program. The *A. aegypti* transcript and the annotated genes are listed along with percentage of sequence similarity between the two. The *Anopheles gambie* orthologs corresponding to these genes, along with gene ontology ID, are also shown to better understand the gene annotation.
(DOC)

Table S3 **Genes that are expressed in correlated manner among the five post infection time points in both the strains.** Two groups of genes (n = 20 and n = 19) are shown that correspond to figure-4 A and B, respectively. The post-infection time points are shown in bold.
(XLSX)

Table S4 **Genes differentially expressed in response to dengue virus infection in a susceptible strain in both the current study and Behura** ***et al*** **., 2011.** The significant transcripts from the present study are listed along with the annotated genes. The fold-change values of expression of these genes are shown both for current study and Behura *et al*., 2011 study, along with the post-infection time points.
(XLSX)

Table S5 **Genes differentially expressed in response to dengue virus infection in a susceptible strain in both the current study and Colpitts** ***et al*** **., 2011, PLoS Pathogens.** The significant transcripts from the present study are listed along with the annotated genes. The fold-change values of expression of these genes are shown both for the current study and Colpitts *et al*., 2011 study, along with the post-infection time points. Note that only 1 day and 2 day post infection time points are common between the two studies.
(XLSX)

Table S6 **Number of significant genes related to different pathways (KEGG).** The list of pathways with significant genes (at least three) identified from the array analysis are shown. The total number of genes annotated by KEGG to the corresponding pathway are also listed for comparison.
(DOCX)

Table S7 **List of primers that were used to perform qRT-PCR assays.** The sequences of forward and reverse primer for each of the cDNAs investigated are listed in 5′–3′ direction.
(DOCX)
