# Supplementary figures and images for: Comparative Expression Profiles of Midgut Genes in Dengue Virus Refractory and Susceptible Aedes aegypti across Critical Period for Virus Infection
Source: PLoS One. 2012 Oct 15;7(10):e47350. doi: 10.1371/journal.pone.0047350 (PMC3471866; doi:10.1371/journal.pone.0047350)

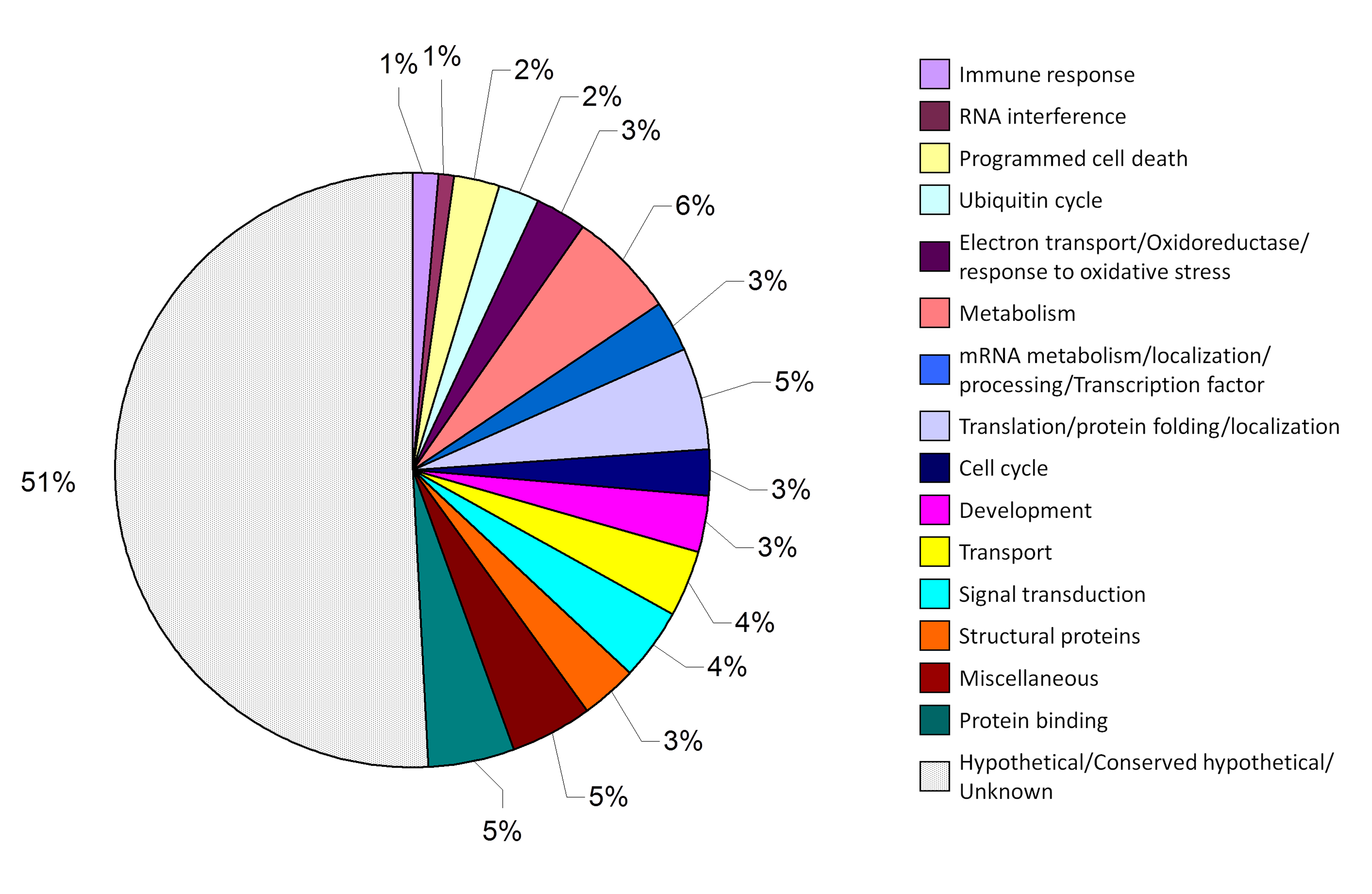

Supplement: Figure S1 — Functional annotations of dengue virus responsive genes. The percentages of responsive genes (in both the strains at all the five time points) are shown for different gene ontology (GO) terms. The GO terms are listed with color codes corresponding to the chart. (TIF) [file pone.0047350.s001.tif]

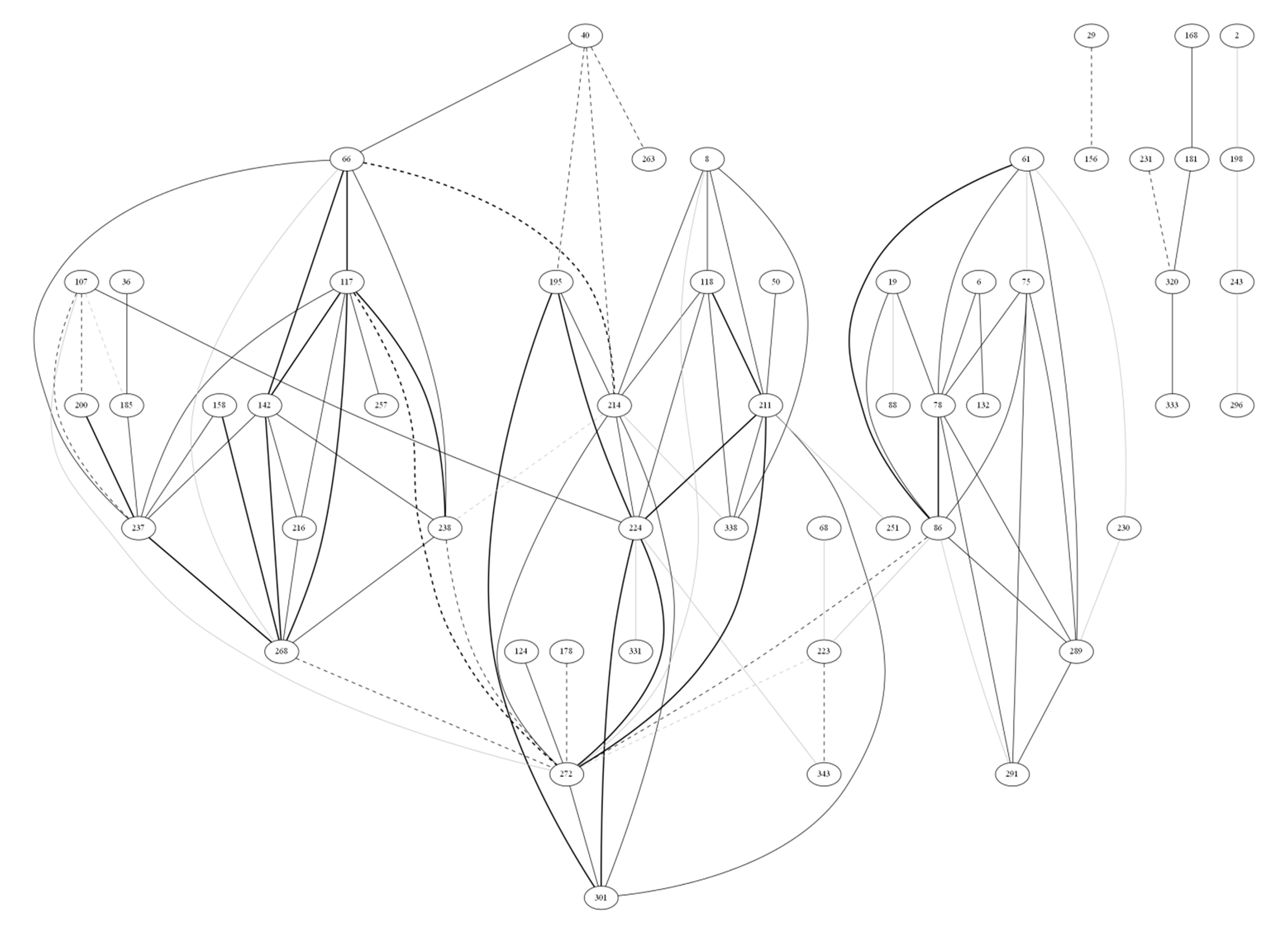

Supplement: Figure S2 — Gene networks of responsive genes that showed highly correlated expression patterns throughout the five post infection time points between susceptible and refractory strains. The nodes of the networks (in circles) represent different genes and the connecting lines represent the interaction patterns. Thickness of individual lines represents either strong or weak interactions determined by partial correlation of expression levels by GenNet software. The network shown here represents only a portion of the entire network. (TIF) [file pone.0047350.s002.tif]
