# Supplementary material for: Comparative Expression Profiles of Midgut Genes in Dengue Virus Refractory and Susceptible Aedes aegypti across Critical Period for Virus Infection
Source: PLoS One. 2012 Oct 15;7(10):e47350. doi: 10.1371/journal.pone.0047350 (PMC3471866; doi:10.1371/journal.pone.0047350)
Supplement: Table S2 — Gene annotations of DETs. Clusters of transcript expression were identified by using the GeneCluster program. The A. aegypti transcript and the annotated genes are listed along with percentage of sequence similarity between the two. The Anopheles gambie orthologs corresponding to these genes, along with gene ontology ID, are also shown to better understand the gene annotation. (DOC) [file pone.0047350.s005.doc]

Table S2. Gene annotations of DETs.

| **Cluster ID** | **EST clone** | 1. ***aegypti*** |  | 1. ***gambiae*** | |  | |
| --- | --- | --- | --- | --- | --- | --- | --- |
|  |  | **Gene ID** | **Gene description** | | **Gene ID** | **Gene description** | **GO ID** |
| 0 | NABNG17 | AAEL009422 | conserved hypothetical protein | | AGAP004864 |  | 5622 |
| 0 | NABNY43 | AAEL010911 | BACH1, putative | |  | diablo | 5515 |
| 0 | NABOL89 | AAEL006667 | phosphatidyltransferase | | AGAP008631 | CG7149 | 8654 |
| 0 | NABPJ55 | AAEL013275 | importin beta-1 | | AGAP009921 | Importin beta subunit | 6607 |
| 0 | NABTY40 | AAEL011240 | hypothetical protein | | AGAP006534 | Z4 | 3676 |
| 0 | NABWN57 | AAEL001061 | glutathione-s-transferase theta, gst | | AGAP004164 | Glutathione S-transferase 1-1 | 4364 |
| 0 | NABX044 | AAEL011302 | annexin | | AGAP012930 | Annexin-B9 | 5544 |
| 0 | NABZ773 | AAEL012673 | ubiquitin conjugating enzyme 7 interacting protein | | AGAP005143 | CG33144 | 5515 |
| 0 | NACBB79 | AAEL012187 | lethal(3)malignant brain tumor | |  |  |  |
| 0 | NADEF37 | AAEL006885 | 14-3-3 protein sigma, gamma, zeta, beta/alpha | | AGAP007643 | 14-3-3-like protein | 8426 |
| 1 | NAAFC23 | AAEL012093 | leucine-rich transmembrane protein | | AGAP006643 | CG5819 |  |
| 1 | NAAG295 | AAEL003349 | NADPH cytochrome P450 | | AGAP000500 | NADPH--cytochrome P450 reductase | 3958 |
| 1 | NAAGN67 | AAEL009217 | mitochondrial ribosomal protein, S35, putative | | AGAP006802 | mitochondrial ribosomal protein S35 | 5515 |
| 1 | NAAGX23 | AAEL005519 | synaptotagmin-14 | | AGAP000430 | CG9778 | 8021 |
| 1 | NABOE34 | AAEL005693 | mitochondrial NADH:ubiquinone oxidoreductase B16.6 subunit, putative | | AGAP009652 | CG3446 |  |
| 1 | NABOV11 | AAEL000859 | hypothetical protein | |  | CG10650 | 5515 |
| 1 | NABPN64 | AAEL002870 | dipeptidyl peptidase iii | | AGAP004394 | Dipeptidyl-peptidase 3 | 17039 |
| 2 | NAAF427 | AAEL006363 | ribose-phosphate pyrophosphokinase 1,2 | | AGAP000954 | CG2246 | 4749 |
| 2 | NAAF645 | AAEL011055 | chaperone protein DNAj | | AGAP007565 | Protein tumorous imaginal discs, mitochondrial precursor | 6986 |
| 2 | NAAF703 | AAEL006061 | proteasome subunit alpha type | | AGAP001995 | Proteasome subunit alpha type 2 | 6511 |
| 2 | NAAF739 | AAEL014768 | glutamate synthase | | AGAP006360 | CG9674 | 6537 |
| 2 | NAAHL61 | AAEL009061 | rrm/rnp domain | | AGAP009601 |  |  |
| 2 | NAAHM86 | AAEL011306 | conserved hypothetical protein | | AGAP004179 | CG4662 | 5509 |
| 2 | NABMS71 | AAEL008388 | ATP-binding cassette sub-family A member 3, putative | | AGAP007504 | CG6052 | 6810 |
| 2 | NABMV08 | AAEL003426 | sodium-dependent phosphate transporter | | AGAP011426 | Na+-dependent inorganic phosphate cotransporter | 15114 |
| 2 | NABN638 | AAEL007625 | conserved hypothetical protein | | AGAP006749 | CG31053 | 5515 |
| 2 | NABO631 | AAEL006260 | serine protease, putative | | AGAP001365 |  |  |
| 2 | NABOE52 | AAEL003494 | goodpasture antigen-binding protein | | AGAP007093 | CG7207 | 5515 |
| 2 | NABPP67 | AAEL010516 | conserved hypothetical protein | | AGAP004023 | CG6194 | 48102 |
| 2 | NABQ703 | AAEL010182 | conserved hypothetical protein | | AGAP003771 | CG1091 | 3676 |
| 2 | NABSV41 | AAEL003129 | neuroligin, | | AGAP003570 | CG31146 | 4759 |
| 2 | NABY667 | AAEL007818 | trypsin | | AGAP008290 | Trypsin 29F | 4252 |
| 2 | NABYB61 | AAEL013652 | oxidoreductase | | AGAP000454 | lethal (2) k14708 | 16491 |
| 2 | NACBM37 | AAEL002879 | heterogeneous nuclear ribonucleoprotein r | | AGAP001419 | CG17838 | 3676 |
| 2 | NACM202 | AAEL015146 | ATP-binding cassette sub-family A member 3, putative | |  |  | 6810 |
| 2 | NADC174 | AAEL006880 | rab32 | | AGAP007654 |  |  |
| 2 | NADCI02 | AAEL008389 | ankyrin repeat-rich membrane-spanning protein | | AGAP012141 | CG30387 | 5515 |
| 2 | NADX465 | AAEL000053 | myotubularin | | AGAP003266 | CG5026 | 4437 |
| 2 | NADY819 | AAEL015410 | AMP deaminase | | AGAP000577 | CG32626 | 9168 |
| 3 | NAAFJ23 | AAEL003539 | WD-repeat protein | | AGAP007739 | F-box-like/WD repeat protein ebi. | 6512 |
| 3 | NAAFL26 | AAEL010665 | developmentally regulated RNA-binding protein | | AGAP005505 | CG1316 | 3676 |
| 3 | NAAFO24 | AAEL011380 | high mobility group B1, putative | | AGAP000005 | High mobility group protein DSP1 | 6268 |
| 3 | NABM343 | AAEL002535 | nucleoprotein, putative | |  |  |  |
| 3 | NABP617 | AAEL009887 | WD-repeat protein | | AGAP001362 | CG1109 |  |
| 3 | NABPX39 | AAEL003661 | translation initiation factor | | AGAP001380 | Probable eukaryotic translation initiation factor 6 | 6413 |
| 3 | NADB983 | AAEL013078 | glycosyltransferase | | AGAP000102 | Probable dolichyl-P-Man:Man(7)GlcNAc(2)-PP-dolichyl-alpha-1,6- mannosyltransferase | 16757 |
| 4 | NAAHQ57 | AAEL008672 | ABC transporter | | AGAP009850 | CG3164 | 16887 |
| 4 | NABMD69 | AAEL004623 | band 4.1-like protein 5, putative | | AGAP004136 |  |  |
| 4 | NABMG18 | AAEL010048 | 26S proteasome non-ATPase regulatory subunit | | AGAP002061 | 26S proteasome non-ATPase regulatory subunit 6 | 5838 |
| 4 | NABOX94 | AAEL001804 | glucosyl/glucuronosyl transferases | | AGAP008401 | CG10178 | 16758 |
| 4 | NABTE93 | AAEL000293 | ebna2 binding protein P100 | | AGAP005672 | Tudor-SN | 4518 |
| 4 | NACAO47 | AAEL007432 | serine collagenase 1 precursor, putative | | AGAP007165 |  |  |
| 5 | NAAHO94 | AAEL010321 | porphobilinogen deaminase | | AGAP011080 | lethal (3) 02640 | 6779 |
| 5 | NAAIB73 | AAEL001389 | conserved hypothetical protein | |  | CG5620 | 8270 |
| 5 | NABMA87 | AAEL005019 | lactosylceramide 4-alpha-galactosyltransferase | | AGAP008258 | 4GT1 | 8378 |
| 5 | NABPA40 | AAEL003006 | 2-deoxyglucose-6-phosphate phosphatase | | AGAP003372 | GS1-like protein. | 8152 |
| 5 | NABPG77 | AAEL001126 | rest corepressor (corest) protein | | AGAP002488 | REST corepressor | 45449 |
| 5 | NABRV48 | AAEL007820 | conserved hypothetical protein | | AGAP002167 | CG15735 | 5515 |
| 5 | NACAK05 | AAEL003572 | RNAse h (70) | | AGAP003847 | CG12877 | 4527 |
| 5 | NACMO33 | AAEL002512 | conserved hypothetical protein | | AGAP000021 | CG2813 | 5515 |
| 5 | NADD680 | AAEL010754 | hypothetical protein | | AGAP012515 | Putative ATP synthase f chain, mitochondrial | 45263 |
| 5 | NADDE53 | AAEL006271 | superoxide dismutase | | AGAP005234 | CG9027 | 6801 |
| 6 | NAAGI05 | AAEL006145 | dynactin | | AGAP004194 | 150 kDa dynein-associated polypeptide | 5856 |
| 6 | NAAGI25 | AAEL010073 | metalloendopeptidase | | AGAP010315 | CG10588 | 4222 |
| 6 | NAAH277 | AAEL008424 | sodium/chloride dependent amino acid transporter | | AGAP010865 | Hypothetical sodium-dependent transporter | 5328 |
| 6 | NAAHV16 | AAEL005944 | mitochondrial ribosomal protein, S23, putative | | AGAP002893 | mRpS23 |  |
| 6 | NABM978 | AAEL011051 | porcupine | | AGAP007567 | CG18445 |  |
| 6 | NABO623 | AAEL004680 | nuclear lamin L1 alpha, putative | | AGAP004237 | CG5376 | 5515 |
| 6 | NABOC41 | AAEL007054 | NADH dehydrogenase, putative | | AGAP012374 | CG10320 | 8137 |
| 6 | NABOI38 | AAEL008750 | conserved hypothetical protein | | AGAP002328 | CG13004 | 5515 |
| 6 | NABOP03 | AAEL012326 | calmodulin | | AGAP012844 | Calmodulin | 5509 |
| 6 | NABPB22 | AAEL008107 | f14p3.9 protein (auxin transport protein) | | AGAP001157 | purity of essence | 4842 |
| 6 | NABPI59 | AAEL008374 | E3 ubiquitin-protein ligase nedd-4 | | AGAP003133 | E3 ubiquitin-protein ligase Nedd-4 | 4842 |
| 6 | NABPQ23 | AAEL002963 | conserved hypothetical protein | | AGAP002461 | CG11791 | 5515 |
| 6 | NABU121 | AAEL007644 | serotonin receptor, putative | | AGAP002679 |  |  |
| 6 | NABXL12 | AAEL003129 | neuroligin, | | AGAP003570 |  | 4759 |
| 6 | NACAG96 | AAEL003950 | helicase | | AGAP010699 |  |  |
| 6 | NACAI24 | AAEL011627 | ribose-5-phosphate isomerase | | AGAP011457 | CG30410 | 9052 |
| 6 | NACAR21 | AAEL013235 | ATP-dependent RNA helicase | | AGAP002829 |  |  |
| 6 | NACAY92 | AAEL005870 | flap endonuclease-1 | | AGAP011448 | Flap endonuclease 1 | 6281 |
| 6 | NACB435 | AAEL001856 | adenosine kinase | | AGAP010136 | Ady43A | 6166 |
| 6 | NACBQ80 | AAEL001112 | conserved hypothetical protein | | AGAP012248 | CG12082 | 6511 |
| 6 | NACMT85 | AAEL014271 | conserved hypothetical protein | | AGAP004631 | CG17271 | 5509 |
| 6 | NADAM61 | AAEL004694 | munc13-4 | | AGAP003164 | CG11819 |  |
| 6 | NADAY41 | AAEL009911 | rotamase | | AGAP004321 | Protein dodo. | 7173 |
| 6 | NADBV14 | AAEL006153 | conserved hypothetical protein | | AGAP002981 | lethal (3) neo43 |  |
| 6 | NADD121 | AAEL001544 | conserved hypothetical protein | | AGAP007614 | Syntaxin Interacting Protein 1 | 8360 |
| 6 | NADDA63 | AAEL004412 | polo kinase kinase | | AGAP010551 | Sterile20-like kinase | 4674 |
| 6 | NADDI72 | AAEL008958 | forkhead box protein (AaegFOXK1) | | AGAP000662 |  |  |
| 6 | NADE575 | AAEL009151 | 30S ribosomal protein S8 | | AGAP000541 | 40S ribosomal protein S15Ab. | 3735 |
| 7 | NAAF052 | AAEL013260 | alpha methylacyl-coa racemase | | AGAP008414 | CG9319 | 8152 |
| 7 | NAAF155 | AAEL005353 | conserved hypothetical protein | | AGAP003357 | CG7272 | 16020 |
| 7 | NAAF222 | AAEL010833 | conserved hypothetical protein | | AGAP006452 | lethal (2) 01289 | 6118 |
| 7 | NAAF310 | AAEL010847 | zinc finger protein | |  |  |  |
| 7 | NAAF389 | AAEL005788 | cytochrome P450 | | AGAP002555 |  |  |
| 7 | NAAF529 | AAEL000193 | histone-lysine n-methyltransferase | | AGAP012481 | Histone-lysine N-methyltransferase, H4 lysine-20 specific | 16571 |
| 7 | NAAF643 | AAEL013144 | eukaryotic translation initiation factor 3 subunit | | AGAP006607 | Eukaryotic translation initiation factor 3 subunit 2 | 3743 |
| 7 | NAAGA36 | AAEL015661 | sterol carrier protein-2, putative | | AGAP004094 | CG12269 |  |
| 7 | NAAGV04 | AAEL011272 | nmda receptor glutamate-binding chain | | AGAP005529 | N-methyl-D-aspartate receptor-associated protein | 4872 |
| 7 | NAAHM83 | AAEL011817 | nonsense-mediated mRNA decay protein 1 (rent1) | | AGAP001133 | Regulator of nonsense transcripts 1 homolog. | 16787 |
| 7 | NABPI87 | AAEL010211 | hypothetical protein | | AGAP001019 | Polycomb protein Pcl | 45892 |
| 7 | NABPL07 | AAEL000608 | conserved hypothetical protein | | AGAP008873 | CG1347 | 5515 |
| 7 | NABPM74 | AAEL008741 | importin (ran-binding protein) | | AGAP010711 | Importin-alpha re-exporter | 6611 |
| 7 | NABPT51 | AAEL008138 | ABC transporter | | AGAP001858 | ABC transporter expressed in trachea | 16887 |
| 7 | NABQ166 | AAEL008159 | short-chain dehydrogenase | | AGAP011852 | CG31546 | 16491 |
| 7 | NABQ939 | AAEL008701 | myoinositol oxygenase | | AGAP003636 | CG6910 |  |
| 7 | NABQF75 | AAEL013554 | cytochrome P450 | |  | Probable cytochrome P450 4ac1 | 16491 |
| 7 | NABRT87 | AAEL012095 | 26S protease regulatory subunit | | AGAP003215 | 26S protease regulatory subunit 4 | 5838 |
| 7 | NABU821 | AAEL012219 | ubiquitin specific protease | | AGAP002900 | CG5798 | 4221 |
| 7 | NABWA11 | AAEL003608 | inositol 1,4,5-trisphosphate receptor | | AGAP006475 | Inositol 1,4,5-trisphosphate receptor | 6816 |
| 7 | NABWT89 | AAEL006523 | crk | | AGAP009499 | Adapter molecule Crk. | 46330 |
| 7 | NACA171 | AAEL012545 | proliferating cell nuclear antigen | | AGAP010220 | CG10262 | 30337 |
| 7 | NACA392 | AAEL004121 | ubiquitin-conjugating enzyme E2 q | | AGAP001452 | CG2924 | 4842 |
| 7 | NACAF55 | AAEL011500 | Cdc42 protein, putative | | AGAP006279 |  |  |
| 7 | NACAF84 | AAEL011317 | actin | | AGAP008687 | Brahma associated protein 55kD | 45893 |
| 7 | NACAP91 | AAEL009192 | serine protease | | AGAP005625 |  |  |
| 7 | NACAQ75 | AAEL014798 | lung carbonyl reductase | | AGAP008502 | CG7322 | 8152 |
| 7 | NACB070 | AAEL004417 | conserved hypothetical protein | | AGAP010074 | CG4663 | 16560 |
| 7 | NACB728 | AAEL000451 | beta1,4 mannosyltransferase | | AGAP003551 | CG18012 | 9058 |
| 7 | NACBO87 | AAEL010202 | trypsin | |  |  | 4252 |
| 7 | NACBQ61 | AAEL014715 | 67 kDa polymerase-associated factor PAF67, putative | | AGAP006130 | CG5642 | 5515 |
| 7 | NACMC73 | AAEL000622 | conserved hypothetical protein | | AGAP009659 | CG6621 | 5515 |
| 7 | NACML07 | AAEL005217 | membrin | | AGAP003800 | Probable Golgi SNAP receptor complex member 2. | 6886 |
| 7 | NACMP24 | AAEL009059 | arp2/3 complex 16 kd subunit (P16-arc) | | AGAP008895 | p16-ARC | 30833 |
| 7 | NACNQ21 | AAEL011308 | serine threonine-protein kinase | | AGAP004176 | ire-1 | 4674 |
| 7 | NADBZ16 | AAEL007326 | ccr4-not transcription complex | | AGAP009057 | CG1884 |  |
| 7 | NADCA90 | AAEL011341 | apyrase, putative | | AGAP007139 | CG1961 | 9166 |
| 7 | NADDC86 | AAEL012064 | Niemann-Pick Type C-2, putative | | AGAP002857 | CG3153 |  |
| 7 | NADDD17 | AAEL001211 | COMPASS component SWD2, putative | | AGAP009700 | CG17293 | 5515 |
| 7 | NADXI31 | AAEL001607 | galactose-1-phosphate uridylyltransferase | | AGAP004451 | Probable galactose-1-phosphate uridylyltransferase | 5975 |
| 7 | NADXJ43 | AAEL009074 | inhibitor of apoptosis 1, diap1 | | AGAP012677 | Apoptosis 1 inhibitor | 43066 |
| 7 | NADYA08 | AAEL012944 | 60S ribosomal protein L11 | | AGAP011173 | 60S ribosomal protein L11. | 3735 |
| 7 | NADZD78 | AAEL014340 | conserved hypothetical protein | | AGAP005243 | Rgk3 | 7264 |
| 8 | NAAFL12 | AAEL005890 | conserved hypothetical protein | | AGAP007534 | Heat shock protein 67B2. | 6986 |
| 8 | NAAGR42 | AAEL009212 | lola | | AGAP005245 | Longitudinals lacking protein, isoforms H/M/V. | 45893 |
| 8 | NAAH040 | AAEL005728 | glycyl-tRNA synthetase | | AGAP008604 | Glycyl-tRNA synthetase | 4812 |
| 8 | NABM809 | AAEL003819 | conserved hypothetical protein | | AGAP011295 | Autophagy-specific gene 1 | 6914 |
| 8 | NABMO84 | AAEL008255 | mbp-1 interacting protein-2a | | AGAP002528 | CG5161 | 6888 |
| 8 | NABNS16 | AAEL006256 | ATPase subunit, putative | | AGAP000523 | CG1746 | 46933 |
| 8 | NABO210 | AAEL011463 | cytochrome P450 | | AGAP005992 | Cytochrome P450 302a1, mitochondrial precursor | 16491 |
| 8 | NABPZ91 | AAEL011453 | galactose-specific C-type lectin, putative | |  |  |  |
| 8 | NABTH12 | AAEL002061 | cation-transporting ATPase 13a1 (g-box binding protein) | | AGAP008085 | CG6230 | 15662 |
| 8 | NABWK75 | AAEL010405 | alkyldihydroxyacetonephosphate synthase | | AGAP004358 | Alkyldihydroxyacetonephosphate synthase | 8610 |
| 8 | NABZE10 | AAEL007177 | conserved hypothetical protein | | AGAP004865 | tungus |  |
| 8 | NACB016 | AAEL014694 | hypothetical protein | |  | CG14303 | 3676 |
| 8 | NACBM70 | AAEL006180 | bumetanide-sensitive Na-K-Cl cotransport protein, putative | | AGAP001557 | sodium chloride cotransporter 69 | 15377 |
| 8 | NADA916 | AAEL008192 | 40S ribosomal protein S3 | | AGAP001910 | 40S ribosomal protein S3. | 3735 |
| 8 | NADBJ43 | AAEL012413 | n-acetyltransferase mak3 | | AGAP003917 | CG11412 | 8080 |
| 8 | NADC615 | AAEL008484 | steroid receptor-interacting snf2 domain protein | | AGAP007573 | CG4049 | 3677 |
| 8 | NADC946 | AAEL011657 | importin alpha | | AGAP001273 | karyopherin 3 | 6606 |
| 8 | NADCD32 | AAEL005567 | nucleosome assembly protein | | AGAP001928 | Nucleosome assembly protein 1 | 42393 |
| 8 | NADCH54 | AAEL009773 | geminin, putative | | AGAP000496 | geminin | 74 |
| 8 | NADCH54 | AAEL004939 | tubulin beta chain | | AGAP004914 | Probable tubulin beta chain. | 7018 |
| 8 | NADCX11 | AAEL004107 | nucleoside diphosphate kinase, putative | | AGAP005123 | nmdyn-D6 | 9209 |
| 8 | NADD746 | AAEL005762 | leucine-rich transmembrane proteins | | AGAP008593 | CG18095 |  |
| 8 | NADD817 | AAEL012980 | conserved hypothetical protein | | AGAP007112 | CG4364 | 8283 |
| 8 | NADDS93 | AAEL003522 | protein arginine n-methyltransferase | | AGAP008846 | Arginine methyltransferase 8 | 8757 |
| 8 | NADDV89 | AAEL008958 | forkhead box protein (AaegFOXK1) | | AGAP000662 |  |  |
| 8 | NADDW93 | AAEL012943 | 26S protease regulatory subunit | | AGAP011174 | Rpt1 | 5838 |
| 8 | NADE926 | AAEL003469 | NHP2 protein, putative | | AGAP012204 | H/ACA ribonucleoprotein complex subunit 2-like protein | 5732 |
| 8 | NADE996 | AAEL007271 | basic helix-loop-helix zip transcription factor | | AGAP009806 | Mlx interactor | 45449 |
| 9 | NAAGO47 | AAEL007562 | conserved hypothetical protein | | AGAP009977 | moladietz | 8105 |
| 9 | NAAI041 | AAEL005583 | c3f | | AGAP012258 | Transmembrane protein nessy. | 16021 |
| 9 | NABM070 | AAEL007106 | serine protease, putative | |  |  |  |
| 9 | NABMS54 | AAEL008319 | protein disulfide isomerase | | AGAP010217 | CG9302 | 16853 |
| 9 | NABO931 | AAEL013239 | bone morphogenetic protein | | AGAP003702 |  |  |
| 9 | NABOA44 | AAEL004351 | casein kinase | | AGAP003997 | gilgamesh | 4674 |
| 9 | NABW743 | AAEL009859 | nucleolar GTP-binding protein | | AGAP007050 | Probable nucleolar GTP-binding protein 1. | 5525 |
| 9 | NACA369 | AAEL006836 | dihydropteridine reductase | | AGAP002534 | Dihydropteridine reductase | 16491 |
| 9 | NADDS62 | AAEL008865 | oligoribonuclease, mitochondrial | | AGAP012804 | Probable oligoribonuclease | 4527 |
| 9 | NADZZ34 | AAEL012301 | hypothetical protein | | AGAP000247 | CG17600 | 5509 |
| 10 | NAAF219 | AAEL005741 | forkhead protein/ forkhead protein domain | | AGAP008606 |  |  |
| 10 | NAAF314 | AAEL004435 | kynurenine aminotransferase | | AGAP011158 | CG6950 | 9058 |
| 10 | NAAF369 | AAEL006794 | dicer-1 | | AGAP012289 | Dicer-2 | 16246 |
| 10 | NAAF704 | AAEL005619 | defective proboscis extension response, putative | | AGAP001201 | dpr7 |  |
| 10 | NAAGH51 | AAEL006425 | trypsin | | AGAP008290 |  | 4252 |
| 10 | NAAHU69 | AAEL006171 | n-myc downstream regulated | | AGAP003238 | CG2082 | 30154 |
| 10 | NABOF96 | AAEL010670 | lethal(2)essential for life protein, l2efl | | AGAP005547 | Heat shock protein 26. | 6986 |
| 10 | NABPK41 | AAEL003327 | zinc finger protein | | AGAP009066 | Zinc-finger protein ush | 6355 |
| 10 | NABPN46 | AAEL001666 | conserved hypothetical protein | | AGAP007823 | CG17018 | 166 |
| 10 | NABX302 | AAEL012556 | Ofd1 protein, putative | | AGAP007857 | CG4213 | 5515 |
| 10 | NABY316 | AAEL007432 | serine collagenase 1 precursor, putative | | AGAP007165 |  |  |
| 10 | NACA373 | AAEL009603 | conserved hypothetical protein | | AGAP006124 | CG32281 | 8757 |
| 10 | NACAF29 | AAEL012064 | Niemann-Pick Type C-2, putative | | AGAP002857 |  |  |
| 10 | NACAJ61 | AAEL012897 | aconitase, mitochondrial | | AGAP007852 | Aconitase | 6099 |
| 10 | NACBP37 | AAEL005782 | conserved hypothetical protein | | AGAP000762 | CG7705 | 5515 |
| 10 | NADDG77 | AAEL011656 | 40S ribosomal protein S15 | | AGAP001274 | Ribosomal protein S15 | 3735 |
| 10 | NADDY03 | AAEL004838 | conserved hypothetical protein | | AGAP006601 | CG1244 | 3676 |
| 10 | NADE078 | AAEL002194 | uricase | | AGAP008440 | Uricase | 6144 |
| 10 | NADXJ88 | AAEL014303 | neuroligin, | | AGAP002090 | neuroligin | 3824 |
| 10 | NADXU73 | AAEL005866 | conserved hypothetical protein | | AGAP012191 | CG12895 | 5515 |
| 11 | NAAFI63 | AAEL005137 | tetraspanin, putative | | AGAP011342 | Tetraspanin 74F | 16021 |
| 11 | NAAG991 | AAEL010245 | chloride channel, putative | | AGAP011245 | icln | 6821 |
| 11 | NAAHL91 | AAEL011309 | orotidine-5'-phosphate decarboxylase, putative | | AGAP004175 | Uridine 5'-monophosphate synthase | 6207 |
| 11 | NAAI190 | AAEL001048 | short-chain dehydrogenase | | AGAP003984 | CG10425 | 8152 |
| 11 | NABOE67 | AAEL013012 | brefeldin a-inhibited guanine nucleotide-exchange protein | | AGAP008906 | CG7578 | 5488 |
| 11 | NABPB77 | AAEL011865 | conserved hypothetical protein | |  | CG11652 | 5515 |
| 11 | NABPC49 | AAEL000153 | conserved hypothetical protein | | AGAP003513 | DOMON domain-containing protein | 6584 |
| 11 | NABPU20 | AAEL007880 | ornithine decarboxylase | | AGAP011805 | Ornithine decarboxylase 1 | 6596 |
| 11 | NABQ789 | AAEL012143 | caspase-1 | | AGAP000830 | Caspase-1 precursor | 6915 |
| 11 | NABQB95 | AAEL005435 | mitochondrial processing peptidase beta subunit | | AGAP000935 | CG3731 | 4222 |
| 11 | NABQD74 | AAEL005985 | conserved hypothetical protein | | AGAP005715 | CG7331 | 5515 |
| 11 | NABW889 | AAEL011387 | leucine-rich repeat | | AGAP000601 | Protein halfway precursor | 7242 |
| 11 | NABWS17 | AAEL004320 | WOC protein, putative | | AGAP001140 | without children | 6697 |
| 11 | NABX337 | AAEL005134 | arylsulfatase b | | AGAP011347 | CG32191 | 8152 |
| 11 | NABXZ42 | AAEL011990 | carbohydrate sulfotransferase | | AGAP009809 | CG31637 | 8146 |
| 11 | NACB983 | AAEL011117 | histone deacetylase | | AGAP001143 | Hdac3 | 16575 |
| 11 | NADBG06 | AAEL001791 | conserved hypothetical protein | | AGAP008397 | CG10283 | 5515 |
| 11 | NADCU87 | AAEL000828 | vitellogenin,, putative | |  | CG5665 | 6629 |
| 11 | NADDH71 | AAEL005931 | 6-phosphogluconate dehydrogenase | | AGAP004197 | 6-phosphogluconate dehydrogenase, decarboxylating | 6098 |
| 12 | NAAF087 | AAEL009047 | hypothetical protein | | AGAP007018 | vismay | 6355 |
| 12 | NAAF122 | AAEL011103 | centromere/kinetochore protein zw10 | | AGAP004467 | Centromere/kinetochore protein zw10 | 51301 |
| 12 | NAAF395 | AAEL001705 | odorant response protein ODR-4, putative | | AGAP006119 | CG10616 | 5515 |
| 12 | NAAF452 | AAEL007704 | lipoma preferred partner/lpp | | AGAP009503 | Zyx102EF | 8270 |
| 12 | NAAF550 | AAEL004716 | chromodomain helicase DNA binding protein | | AGAP008698 | Chromodomain-helicase-DNA-binding protein | 6333 |
| 12 | NAAFC38 | AAEL000101 | AMP dependent coa ligase | |  |  |  |
| 12 | NAAFH31 | AAEL007383 | secreted ferritin G subunit precursor, putative | | AGAP002464 | Ferritin 2 light chain homologue | 6879 |
| 12 | NAAFQ07 | AAEL010832 | Pop5 protein, putative | | AGAP005957 | CG14057 | 8033 |
| 12 | NAAGI67 | AAEL002554 | anosmin, putative | | AGAP003441 |  |  |
| 12 | NAAGY29 | AAEL013279 | peptidyl-prolyl cis-trans isomerase (cyclophilin) | | AGAP007088 | CG2852 | 6457 |
| 12 | NAAH081 | AAEL006547 | THO complex, putative | | AGAP005429 | thoc6 |  |
| 12 | NAAHE09 | AAEL008963 | tyrosine aminotransferase | | AGAP000327 | CG1461 | 6519 |
| 12 | NAAHL85 | AAEL007913 | sulfotransferase (sult) | | AGAP012672 |  |  |
| 12 | NAAHR02 | AAEL011145 | ribosomal protein S6 kinase, 90kD, polypeptide | |  |  |  |
| 12 | NAAI573 | AAEL003722 | ribonuclease UK114, putative | | AGAP005327 | CG15261 |  |
| 12 | NAAI761 | AAEL006613 | pickpocket | | AGAP007945 | pickpocket 13 | 5272 |
| 12 | NAAI837 | AAEL014301 | hypothetical protein | | AGAP002736 | raw | 48102 |
| 12 | NAAIE80 | AAEL008848 | ATP synthase gamma subunit | | AGAP007966 | ATP synthase gamma chain, mitochondrial precursor | 15986 |
| 12 | NABM551 | AAEL011197 | actin | | AGAP000651 | Actin-42A. | 5198 |
| 12 | NABM741 | AAEL003973 | conserved hypothetical protein | | AGAP012061 | CG11166 | 5515 |
| 12 | NABMA02 | AAEL006472 | rabconnectin | | AGAP010490 | CG3585 | 6810 |
| 12 | NABMB46 | AAEL001567 | myocyte-specific enhancer factor 2d | | AGAP007608 | Myocyte-specific enhancer factor 2 | 7519 |
| 12 | NABMD03 | AAEL005238 | mck1 | | AGAP004443 | Protein kinase shaggy | 16055 |
| 12 | NABMV42 | AAEL004514 | conserved hypothetical protein | | AGAP000615 | CG10221 | 5488 |
| 12 | NABMY03 | AAEL007994 | conserved hypothetical protein | |  | CG11539 | 8080 |
| 12 | NABN733 | AAEL011291 | protease m1 zinc metalloprotease | | AGAP000885 | CG11951 | 4179 |
| 12 | NABNS81 | AAEL001356 | RNA-binding protein | | AGAP007325 | RNA-binding protein 1. | 6397 |
| 12 | NABO173 | AAEL006021 | hypothetical protein | | AGAP004795 | Su(Tpl) |  |
| 12 | NABOF14 | AAEL003245 | Inhibitor of nuclear factor kappa B kinase beta subunit | | AGAP009166 |  |  |
| 12 | NABOH80 | AAEL003911 | conserved hypothetical protein | | AGAP002386 | CG14650 | 51082 |
| 12 | NABOK62 | AAEL008490 | NADH dehydrogenase, putative | | AGAP007574 | CG5548 | 6118 |
| 12 | NABOO95 | AAEL001421 | high density lipoprotien binding protein / vigilin | | AGAP005467 | Dodeca-satellite-binding protein 1 | 792 |
| 12 | NABP555 | AAEL002284 | mediator complex, subunit, putative | | AGAP010936 | Mediator complex subunit 18 | 5515 |
| 12 | NABP658 | AAEL007457 | insect origin recognition complex subunit | | AGAP000474 | Origin recognition complex subunit 2 | 6270 |
| 12 | NABPB36 | AAEL005425 | conserved hypothetical protein | | AGAP003715 | auxillin | 16191 |
| 12 | NABPB87 | AAEL008863 | protein regulator of cytokinesis 1 prc1 | | AGAP012520 | fascetto | 910 |
| 12 | NABPK86 | AAEL007211 | conserved hypothetical protein | | AGAP003417 | CG8449 | 5515 |
| 12 | NABPL28 | AAEL015309 | actin | | AGAP002127 | Actin-like protein 87C. | 5198 |
| 12 | NABPP03 | AAEL014493 | aldehyde oxidase | | AGAP007918 | Xanthine dehydrogenase | 6118 |
| 12 | NABPQ03 | AAEL011817 | nonsense-mediated mRNA decay protein 1 (rent1) | | AGAP001133 |  | 4386 |
| 12 | NABPU10 | AAEL002269 | purine nucleoside phosphorylase | | AGAP005944 | CG18128 | 4731 |
| 12 | NABQD40 | AAEL011993 | conserved hypothetical protein | | AGAP008836 | mus81 | 6259 |
| 12 | NABQD75 | AAEL001807 | cytochrome P450 | | AGAP009363 | Cytochrome P450 9b1 | 16491 |
| 12 | NABR452 | AAEL007331 | attractin | | AGAP003506 | distracted | 5198 |
| 12 | NABRF06 | AAEL008716 | conserved hypothetical protein | | AGAP012084 | CG13773 | 3899 |
| 12 | NABRS77 | AAEL008696 | smad | | AGAP009777 | Smad on X | 6355 |
| 12 | NABS886 | AAEL005027 | acidic ribosomal protein P1, putative | | AGAP007740 | 60S acidic ribosomal protein P1 | 3735 |
| 12 | NABSA62 | AAEL005325 | dopachrome-conversion enzyme (DCE) isoenzyme, putative | | AGAP007549 | yellow-h |  |
| 12 | NABSC39 | AAEL001703 | serine-type enodpeptidase, | |  |  |  |
| 12 | NABSD20 | AAEL011708 | heat shock protein | | AGAP006958 | Heat shock protein 83 | 6986 |
| 12 | NABSH78 | AAEL004941 | cytochrome P450 | | AGAP010961 | Probable cytochrome P450 6w1 | 16491 |
| 12 | NABSX40 | AAEL013138 | conserved hypothetical protein | | AGAP012008 | SRY interacting protein 1 | 5515 |
| 12 | NABTT85 | AAEL004035 | importin 11 (imp11) (ran-binding protein 11) | | AGAP002970 | Ranbp11 | 59 |
| 12 | NABUW32 | AAEL000642 | alpha-amylase | | AGAP012399 | Probable maltase D precursor | 16798 |
| 12 | NABUW82 | AAEL008958 | forkhead box protein (AaegFOXK1) | | AGAP000662 |  |  |
| 12 | NABUZ18 | AAEL006860 | ribosomal protein S28, putative | | AGAP003412 | Ribosomal protein S28a | 3735 |
| 12 | NABW176 | AAEL007762 | mitochondrial ribosomal protein, L40, putative | | AGAP000110 | mitochondrial ribosomal protein L40 | 5515 |
| 12 | NABW221 | AAEL007699 | 60S ribosomal protein L9 | | AGAP009508 | 60S ribosomal protein L9. | 3735 |
| 12 | NABW851 | AAEL003203 | fatty acid desaturase, putative | | AGAP012920 | Fad2 | 6633 |
| 12 | NABWG55 | AAEL013131 | hypothetical protein | | AGAP012023 | Protein chiffon. | 6260 |
| 12 | NABWW36 | AAEL009306 | conserved hypothetical protein | | AGAP007117 | CG13567 | 5515 |
| 12 | NABWX07 | AAEL000178 | ubiquinone/menaquinone biosynthesis methyltransferase | | AGAP010488 | Ubiquinone biosynthesis methyltransferase COQ5, mitochondrial precursor | 9108 |
| 12 | NABWY63 | AAEL014068 | conserved hypothetical protein | | AGAP009081 | CG10470 | 5515 |
| 12 | NABWZ81 | AAEL010576 | modifier of mdg4 | | AGAP003439 | Modifier of mdg4. | 1672 |
| 12 | NABXG70 | AAEL014550 | homeobox protein pknox1 | | AGAP007539 |  |  |
| 12 | NABXX91 | AAEL007765 | serine protease inhibitor 4, serpin-4 | | AGAP005246 | CG6717 | 4867 |
| 12 | NABXY34 | AAEL010605 | zinc finger protein | |  | CG10274 | 3676 |
| 12 | NABY175 | AAEL004389 | mannosidase alpha class 2a | | AGAP004032 | -Man-IIb | 5975 |
| 12 | NABY770 | AAEL010396 | secreted ferritin G subunit precursor, putative | | AGAP002464 |  | 6879 |
| 12 | NABZ375 | AAEL013863 | phosphatidylinositol-binding clathrin assembly protein | | AGAP010087 | Phosphatidylinositol-binding clathrin assembly protein LAP | 6898 |
| 12 | NACA170 | AAEL004570 | conserved hypothetical protein | | AGAP001400 | CG31109 | 8270 |
| 12 | NACA527 | AAEL006830 | yellow protein precursor | | AGAP000879 |  |  |
| 12 | NACAL40 | AAEL006727 | multisynthetase complex, auxiliary protein, p38, putative | | AGAP005418 | Probable multisynthetase complex auxiliary component p38. | 6412 |
| 12 | NACAL70 | AAEL006776 | blastoderm specific protein 25D, putative | | AGAP010445 | CG33991 | 31532 |
| 12 | NACAM05 | AAEL009635 | conserved hypothetical protein | | AGAP004537 | CG13886 | 5515 |
| 12 | NACB817 | AAEL008592 | ribonuclease iii | | AGAP008087 | drosha | 31053 |
| 12 | NACBA41 | AAEL013530 | cullin | | AGAP008007 | Cullin homolog 1 | 19005 |
| 12 | NACBR21 | AAEL012840 | stathmin | | AGAP009940 | stathmin | 7242 |
| 12 | NACBV28 | AAEL014749 | ral | | AGAP012108 | Ras-related protein Ral-a. | 46329 |
| 12 | NACM460 | AAEL007684 | tektin, putative | | AGAP010031 | Tektin A | 226 |
| 12 | NACMW60 | AAEL005160 | sulphate transporter | | AGAP002331 | Epidermal stripes and patches | 5215 |
| 12 | NACNJ41 | AAEL010366 | glucosyl/glucuronosyl transferases | | AGAP007028 |  |  |
| 12 | NADAI49 | AAEL007240 | cdc42 GTPase-activating protein | | AGAP009303 | CdGAPr | 5096 |
| 12 | NADAU20 | AAEL002881 | NADH:ubiquinone dehydrogenase, putative | | AGAP003325 | CG32230 |  |
| 12 | NADB267 | AAEL003237 | low molecular weight protein-tyrosine-phosphatase | | AGAP009266 | CG31469 | 6470 |
| 12 | NADB479 | AAEL005833 | cytosolic purine 5-nucleotidase | | AGAP000380 | CG32549 |  |
| 12 | NADB789 | AAEL007518 | complexin, putative | | AGAP003509 | complexin | 6836 |
| 12 | NADBF79 | AAEL007390 | UDP-glucose 4-epimerase | | AGAP012261 | Probable UDP-glucose 4-epimerase | 6012 |
| 12 | NADBI20 | AAEL014440 | juvenile hormone-inducible protein, putative | |  |  |  |
| 12 | NADC862 | AAEL008461 | surfeit locus protein | | AGAP001069 | Surfeit locus protein 4 homolog. | 5783 |
| 12 | NADCV84 | AAEL007113 | selenophosphate synthase | | AGAP011375 | Selenide, water dikinase 2 | 16260 |
| 12 | NADD117 | AAEL001005 | calreticulin | | AGAP004212 | Calreticulin precursor | 5509 |
| 12 | NADD559 | AAEL014028 | hypothetical protein | | AGAP005731 | Transcription initiation factor TFIID subunit 2 | 4179 |
| 12 | NADD585 | AAEL008738 | DEAD box ATP-dependent RNA helicase | | AGAP012045 |  |  |
| 12 | NADD987 | AAEL012057 | enhancer of polycomb | | AGAP008026 | Enhancer of Polycomb | 16573 |
| 12 | NADDA34 | AAEL004278 | conserved hypothetical protein | | AGAP010150 | Cytochrome b5-related protein | 16491 |
| 12 | NADDE33 | AAEL006389 | cathepsin l | | AGAP012577 | 26-29kD-proteinase | 4197 |
| 12 | NADDF51 | AAEL008678 | conserved hypothetical protein | | AGAP005829 | CG32371 | 8017 |
| 12 | NADDK16 | AAEL006256 | ATPase subunit, putative | | AGAP000523 |  | 15986 |
| 12 | NADDW16 | AAEL007914 | discs large protein | |  |  |  |
| 12 | NADE237 | AAEL006939 | smaug protein | |  | Protein Smaug. | 900 |
| 12 | NADE879 | AAEL000970 | predicted protein | | AGAP011583 | Transcription factor GAGA | 45893 |
| 12 | NADEA62 | AAEL004897 | brain chitinase and chia | | AGAP006898 | CG1869 | 6030 |
| 12 | NADEF11 | AAEL013522 | pinn | | AGAP003316 | Pinin | 5515 |
| 12 | NADVX13 | AAEL001235 | palmitoyl-protein thioesterase | | AGAP009695 | CG4851 | 8474 |
| 13 | NAAF408 | AAEL007088 | ribitol kinase | | AGAP005956 | CG11594 | 5975 |
| 13 | NAAFN07 | AAEL003868 | DNA repair protein xp-c / rad4 | | AGAP003345 | DNA-repair protein complementing XP-C cells homolog | 6974 |
| 13 | NAAFQ19 | AAEL013623 | trypsin | |  |  |  |
| 13 | NAAFS21 | AAEL001160 | zinc finger protein | |  |  |  |
| 13 | NAAFT02 | AAEL002581 | structural maintenance of chromosomes 6 smc6 | | AGAP002985 | CG5524 | 51276 |
| 13 | NAAFV04 | AAEL008668 | MASP-2 protein, putative | |  |  |  |
| 13 | NAAHT60 | AAEL006870 | sorting nexin | | AGAP011491 | CG5734 | 7242 |
| 13 | NAAI590 | AAEL003801 | 28S ribosomal protein S5 | | AGAP004091 | CG40049 | 3735 |
| 13 | NAAI753 | AAEL003946 | mitochondrial ribosomal protein S33, putative | | AGAP002777 | mitochondrial ribosomal protein S33 |  |
| 13 | NAAIC41 | AAEL013981 | hexamerin 2 beta | |  | Larval serum protein 1 beta chain precursor | 5344 |
| 13 | NAAIC81 | AAEL003594 | kinectin, putative | |  |  |  |
| 13 | NABMD09 | AAEL014034 | zinc finger protein | | AGAP000668 |  |  |
| 13 | NABMV71 | AAEL001969 | protein serine/threonine kinase, putative | | AGAP008054 | Ejaculatory bulb-specific protein 3 precursor | 5515 |
| 13 | NABN118 | AAEL009831 | pyrroline-5-carboxylate reductase | |  | CG5840 | 6561 |
| 13 | NABNA40 | AAEL013147 | hypothetical protein | | AGAP005162 | Dystroglycan | 5509 |
| 13 | NABNQ53 | AAEL007337 | ubiquitin-conjugating enzyme E2 q | | AGAP008575 | CG4502 | 4842 |
| 13 | NABOK41 | AAEL003419 | conserved hypothetical protein | | AGAP011936 | CG32209 | 6030 |
| 13 | NABOS72 | AAEL006481 | conserved hypothetical protein | | AGAP001184 | CheA7a |  |
| 13 | NABP386 | AAEL003206 | glutaredoxin, grx | | AGAP003415 | CG6523 | 45454 |
| 13 | NABP880 | AAEL010373 | dullard protein | | AGAP006231 | lethal (1) G0269 | 16791 |
| 13 | NABPA73 | AAEL002501 | protein disulfide isomerase | | AGAP000044 | CG1837 | 16853 |
| 13 | NABPD69 | AAEL013733 | Psq-DNA binding domain protein, putative | |  |  |  |
| 13 | NABPD91 | AAEL000418 | conserved hypothetical protein | | AGAP002730 | lethal (1) G0136 | 5515 |
| 13 | NABPM80 | AAEL005410 | erythroblast macrophage protein emp | | AGAP004332 | CG31357 | 5515 |
| 13 | NABPN38 | AAEL008160 | fatty acid synthase | | AGAP001899 | CG17374 | 6633 |
| 13 | NABPU48 | AAEL009623 | RNA 3' terminal phosphate cyclase | | AGAP004820 | RNA 3'-terminal phosphate cyclase | 3963 |
| 13 | NABPV26 | AAEL014019 | cytochrome P450 | | AGAP006047 |  | 16491 |
| 13 | NABPZ90 | AAEL005009 | groucho protein (enhancer of split) | | AGAP010324 |  |  |
| 13 | NABQ611 | AAEL002286 | Autophagy-specific protein, putative | | AGAP010939 | Autophagy protein 5 | 48102 |
| 13 | NABRN58 | AAEL002324 | conserved hypothetical protein | | AGAP005344 | VPS28 protein homolog. | 15031 |
| 13 | NABRV03 | AAEL001549 | protein kinase c | |  |  |  |
| 13 | NABRV30 | AAEL001847 | conserved hypothetical protein | | AGAP007778 | CG4644 | 3899 |
| 13 | NABSD43 | AAEL011657 | importin alpha | | AGAP001273 |  | 6606 |
| 13 | NABSE14 | AAEL011713 | conserved hypothetical protein | | AGAP005921 | CG10581 | 103 |
| 13 | NABSV81 | AAEL007796 | serine protease | | AGAP002422 | CG9372 | 4252 |
| 13 | NABUH83 | AAEL008198 | EGF repeat molecule, putative | | AGAP007256 | draper | 6909 |
| 13 | NABWF78 | AAEL007103 | p37NB protein, putative | | AGAP007045 | CG4950 |  |
| 13 | NABWX40 | AAEL002331 | mannose-6-phosphate isomerase | | AGAP000053 | CG8417 | 5975 |
| 13 | NABX402 | AAEL011921 | conserved hypothetical protein | | AGAP000471 | CG1839 | 6512 |
| 13 | NABYB68 | AAEL009913 | DEAD box ATP-dependent RNA helicase | | AGAP003397 | DEAD-box helicase Dbp80 | 3723 |
| 13 | NABYD94 | AAEL011863 | ABC transporter | |  | CG32091 | 16887 |
| 13 | NACAG25 | AAEL006776 | blastoderm specific protein 25D, putative | | AGAP010445 |  | 31532 |
| 13 | NACBI51 | AAEL010576 | modifier of mdg4 | | AGAP003439 |  | 42981 |
| 13 | NACBV90 | AAEL009084 | slender lobes, putative | | AGAP007295 | CG5645 | 5515 |
| 13 | NACMW32 | AAEL014233 | pickpocket | |  |  |  |
| 13 | NADB107 | AAEL000217 | serine/threonine protein kinase | | AGAP003201 | Tao-1 | 6468 |
| 13 | NADBI77 | AAEL000028 | serine protease | | AGAP012614 | CG16705 | 4252 |
| 13 | NADCH04 | AAEL000641 | protein disulfide isomerase | | AGAP012407 | Protein disulfide-isomerase precursor | 16853 |
| 13 | NADCP27 | AAEL007271 | basic helix-loop-helix zip transcription factor | | AGAP009806 |  | 45449 |
| 13 | NADDG82 | AAEL014142 | phosphatidylinositol synthase | | AGAP003478 | CG9245 | 8654 |
| 13 | NADDT83 | AAEL006189 | conserved hypothetical protein | | AGAP004489 | CG8814 | 62 |
| 13 | NADDU56 | AAEL004249 | conserved hypothetical protein | | AGAP007249 | Flightin | 30016 |
| 13 | NADE381 | AAEL011099 | molybdopterin-binding | | AGAP002740 | CG16848 | 8152 |
| 13 | NADEC28 | AAEL002411 | conserved hypothetical protein | | AGAP003430 | CG8444 | 4872 |
| 13 | NADWR53 | AAEL011813 | conserved hypothetical protein | | AGAP001136 | CG3678 | 5515 |
| 14 | NAAF909 | AAEL006409 | sialin, sodium/sialic acid cotransporter, putative | | AGAP008942 | CG15438 | 5215 |
| 14 | NAAG452 | AAEL003165 | low molecular weight protein-tyrosine-phosphatase | | AGAP004079 | Low molecular weight phosphotyrosine protein phosphatase 1 | 4721 |
| 14 | NAAGJ40 | AAEL002214 | amino acid transporter | | AGAP008490 | CG13384 | 6865 |
| 14 | NABMC48 | AAEL003148 | short-chain dehydrogenase | | AGAP011357 |  |  |
| 14 | NABML88 | AAEL004125 | signal transduction protein lnk-realted | | AGAP003863 | Lnk | 7242 |
| 14 | NABMN26 | AAEL009047 | hypothetical protein | | AGAP007018 |  | 6355 |
| 14 | NABMN54 | AAEL008697 | cytochrome c oxidase, subunit VB, putative | | AGAP008724 | CG11015 | 6118 |
| 14 | NABOD15 | AAEL010018 | hypothetical protein | | AGAP004937 | CG7015 | 6355 |
| 14 | NABPW84 | AAEL004284 | mitochondrial ATPase inhibitor, putative | | AGAP008898 | CG13551 | 45978 |
| 14 | NABWQ31 | AAEL005862 | hypothetical protein | |  | Oxysterol binding protein | 8202 |
| 14 | NABX360 | AAEL004237 | vacuolar protein sorting 18 (deep orange protein) | | AGAP000983 | Vacuolar protein sorting 18 | 8333 |
| 14 | NABXC23 | AAEL002084 | suppressor of defective silencing | | AGAP000738 | CG14220 | 5515 |
| 14 | NABYE77 | AAEL006400 | elongator component, putative | | AGAP008300 | CG15433 | 15986 |
| 14 | NABZ392 | AAEL008227 | short-chain dehydrogenase | | AGAP004450 | CG13377 | 16491 |
| 14 | NABZA38 | AAEL004911 | DEAD box ATP-dependent RNA helicase | | AGAP005391 |  |  |
| 14 | NACBG25 | AAEL007687 | transmembrane 9 superfamily protein member 4 | | AGAP010029 | CG7364 | 5215 |
| 14 | NACBQ60 | AAEL002185 | cuticle protein, putative | | AGAP008444 | Adult cuticle protein 1 precursor | 5198 |
| 14 | NACBT91 | AAEL007084 | conserved hypothetical protein | | AGAP005973 | Methyltransferase-like protein | 8757 |
| 14 | NACMR05 | AAEL009891 | conserved hypothetical protein | | AGAP003276 | Xe7 | 5515 |
| 14 | NADAG79 | AAEL012705 | conserved hypothetical protein | | AGAP000886 | CG5196 | 8270 |
| 14 | NADAW91 | AAEL007752 | cytochrome c oxidase, subunit VIIA, putative | | AGAP000109 | Probable cytochrome c oxidase polypeptide VIIa, mitochondrial precursor | 16491 |
| 14 | NADCX10 | AAEL006573 | conserved hypothetical protein | | AGAP006184 | CG8547 | 5515 |
| 14 | NADCX72 | AAEL013305 | bifunctional dihydrofolate reductase-thymidylate synthase | | AGAP010457 | Thymidylate synthase | 9165 |
| 14 | NADXL06 | AAEL007705 | hect E3 ubiquitin ligase | | AGAP009511 | CG5604 | 4842 |
| 14 | NADYL62 | AAEL004974 | beta-1,3-glucuronyltransferase s, p | | AGAP008806 | Galactosylgalactosylxylosylprotein 3-beta-glucuronosyltransferase S | 6688 |
| 15 | NAAGI28 | AAEL000109 | enolase-phosphatase e-1 | |  |  |  |
| 15 | NAAHD59 | AAEL010230 | NADH:ubiquinone dehydrogenase, putative | | AGAP009865 | CG9306 | 5515 |
| 15 | NAAI512 | AAEL010204 | dihydropyrimidine dehydrogenase | | AGAP001021 | Rhythmically expressed gene 3 | 6207 |
| 15 | NABM696 | AAEL015384 | grb2-associated binder, gab | |  | daughter of sevenless | 8293 |
| 15 | NABNN67 | AAEL007322 | phosphatidate phosphatase | | AGAP012445 | Putative phosphatidate phosphatase | 35234 |
| 15 | NABQ552 | AAEL001605 | microtubule binding protein, putative | | AGAP005075 | Mapmodulin |  |
| 15 | NABRI32 | AAEL011098 | conserved hypothetical protein | | AGAP003227 | nmdyn-D7 | 4550 |
| 15 | NACAT82 | AAEL000883 | hypothetical protein | | AGAP011762 | CG32130 | 6915 |
| 15 | NACAU46 | AAEL003957 | conserved hypothetical protein | | AGAP012056 | Cofilin/actin-depolymerizing factor homolog | 7015 |
| 15 | NACBC74 | AAEL013675 | eukaryotic translation initiation factor | | AGAP011190 | Eukaryotic translation initiation factor 2 alpha subunit | 3743 |
| 15 | NADAL09 | AAEL000622 | conserved hypothetical protein | | AGAP009659 |  | 5515 |
| 15 | NADBM61 | AAEL004450 | cytochrome b5, putative | | AGAP002113 | CG5157 | 5506 |
| 15 | NADCA83 | AAEL001859 | vesicle protein sorting-associated | | AGAP011358 | Sly1 protein homolog. | 6904 |
| 15 | NADE693 | AAEL008865 | oligoribonuclease, mitochondrial | | AGAP012804 |  | 4527 |
| 16 | NAAF181 | AAEL009722 | clip-domain serine protease, putative | | AGAP004639 |  |  |
| 16 | NAAG339 | AAEL004688 | conserved hypothetical protein | | AGAP000868 | Beta 1,4-mannosyltransferase egh | 6688 |
| 16 | NAAH353 | AAEL008638 | cytochrome P450 | | AGAP005774 | Probable cytochrome P450 49a1 | 16491 |
| 16 | NAAI815 | AAEL005319 | myosin light chain kinase | |  |  |  |
| 16 | NABMF05 | AAEL003913 | methionine-tRNA synthetase | | AGAP002383 | Methionyl-tRNA synthetase, mitochondrial precursor | 6418 |
| 16 | NABNI58 | AAEL011436 | myosin xv | | AGAP005213 | unconventional myosin class XV | 3774 |
| 16 | NABOO31 | AAEL007658 | partitioning defective 3, par-3 | | AGAP000869 | bazooka | 8356 |
| 16 | NABPD27 | AAEL002673 | elongase, putative | | AGAP008780 | CG5278 | 16021 |
| 16 | NABPI14 | AAEL004235 | kinesin-like protein Klp10A | | AGAP000159 | Kinesin-like protein Klp10A | 5828 |
| 16 | NABPM17 | AAEL014931 | sarm1 | | AGAP005901 | Sterile alpha and TIR motif-containing protein 1 | 45087 |
| 16 | NABPP84 | AAEL013567 | ATP-dependent bile acid permease | | AGAP006427 | CG7627 | 42626 |
| 16 | NABW043 | AAEL001963 | protein serine/threonine kinase, putative | | AGAP008059 | Pherokine 3 | 7265 |
| 16 | NABWE19 | AAEL011091 | retinal degeneration b beta | | AGAP004469 | rdgB | 6810 |
| 16 | NABWO52 | AAEL008183 | t complex protein | | AGAP001897 | lethal (3) s2214 | 5515 |
| 16 | NACA489 | AAEL002597 | conserved hypothetical protein | | AGAP011797 | CG11009 | 5515 |
| 16 | NACAC14 | AAEL006133 | cofactor A, putative | | AGAP002130 | CG1890 | 51082 |
| 16 | NACAM48 | AAEL013979 | conserved hypothetical protein | | AGAP011108 | GXIVsPLA2 | 16042 |
| 16 | NACBI10 | AAEL013907 | d-alanyl-d-alanine carboxypeptidase | | AGAP011533 | CG11396 | 5488 |
| 16 | NACBS65 | AAEL005455 | CTP synthase | | AGAP009624 | CG6854 | 6221 |
| 16 | NADBI46 | AAEL001447 | conserved hypothetical protein | | AGAP000456 | CG6982 | 5198 |
| 16 | NADBJ96 | AAEL014042 | protein phosphatase pp2a regulatory subunit b | | AGAP004502 | Protein phosphatase PP2A 55 kDa regulatory subunit | 16055 |
| 16 | NADBK44 | AAEL008507 | srpk | |  |  |  |
| 16 | NADC566 | AAEL000900 | conserved hypothetical protein | | AGAP010736 | CG8247 | 7349 |
| 16 | NADCB55 | AAEL011114 | serine/threonine-protein kinase rio2 (rio kinase 2) | | AGAP001526 | CG11859 | 16301 |
| 16 | NADCE53 | AAEL001129 | conserved hypothetical protein | | AGAP002501 | Protein phosphatase 4 regulatory subunit 2-related protein |  |
| 16 | NADCP92 | AAEL002508 | 26S protease regulatory subunit 6a | | AGAP000616 | Tat-binding protein-1 | 5838 |
| 16 | NADE088 | AAEL007066 | mitotic checkpoint protein and poly(a)+ RNA export protein | | AGAP012373 | CG12782 | 5515 |
| 16 | NADE869 | AAEL009762 | cytochrome P450 | | AGAP001039 | Cytochrome P450 307a1 | 16491 |
| 16 | NADX995 | AAEL012026 | translation initiation factor 5C, putative | | AGAP002413 | eukaryotic initiation factor 5C | 3743 |
| 16 | NADY537 | AAEL002469 | endophilin a, | | AGAP004766 | endophilin A | 48488 |
| 17 | NAAFD60 | AAEL004805 | potassium-dependent sodium-calcium exchanger, putative | | AGAP010975 |  |  |
| 17 | NAAHT06 | AAEL010900 | importin alpha | | AGAP005401 | karyopherin 1 | 6606 |
| 17 | NAAI466 | AAEL004496 | glutamate transporter | | AGAP009443 | Excitatory amino acid transporter 1 | 17153 |
| 17 | NABM594 | AAEL003498 | down syndrome cell adhesion molecule | | AGAP007092 | CG33274 | 7275 |
| 17 | NABOI47 | AAEL006207 | conserved hypothetical protein | |  | CG6830 | 16301 |
| 17 | NABPR07 | AAEL010509 | bridging integrator | | AGAP005076 | Amphiphysin | 6887 |
| 17 | NABPV07 | AAEL001371 | pre-mRNA cleavage factor im, 25kD subunit | | AGAP007242 | CG3689 | 5515 |
| 17 | NABQ414 | AAEL005113 | alpha-esterase | | AGAP006725 | cricklet | 4091 |
| 17 | NABQ561 | AAEL003743 | vacuolar proton ATPases | | AGAP003711 | Vha100-1 | 15992 |
| 17 | NABQ747 | AAEL006568 | serine protease | |  |  |  |
| 17 | NABR617 | AAEL008705 | hypothetical protein | | AGAP003637 | proximal to raf |  |
| 17 | NABSW88 | AAEL004565 | ADP-ribosylation factor, arf | | AGAP002931 | ADP-ribosylation factor 2 | 6364 |
| 17 | NABW733 | AAEL005384 | phosphoribosylformylglycinamidine synthase, putative | | AGAP002091 | Phosphoribosylformylglycinamidine synthase | 6164 |
| 17 | NABZB68 | AAEL006751 | hypothetical protein | |  | CG2217 | 5515 |
| 17 | NACAZ73 | AAEL012746 | chaperonin | | AGAP010588 | CG8258 | 51082 |
| 17 | NACNM57 | AAEL013613 | pyruvate dehydrogenase | | AGAP003030 | lethal (1) G0334 | 16624 |
| 17 | NADCF81 | AAEL008952 | sentrin/sumo-specific protease | |  | CG11023 | 8234 |
| 17 | NADDG86 | AAEL011830 | conserved hypothetical protein | | AGAP000470 | CG11134 | 5515 |
| 17 | NADDX56 | AAEL012166 | conserved hypothetical protein | | AGAP005133 | Protein tamozhennic. | 8536 |
| 17 | NADEF81 | AAEL006951 | protein phosphatase 2c | | AGAP002141 | CG12091 | 3824 |
| 17 | NADYE59 | AAEL003586 | neuronal cell adhesion molecule | | AGAP008943 | CG16857 | 7155 |
| 18 | NAAF257 | AAEL009131 | cytochrome P450 | |  | Probable cytochrome P450 6d2 | 16491 |
| 18 | NAAH539 | AAEL012825 | bifunctional purine biosynthesis protein | | AGAP001423 | CG11089 | 6164 |
| 18 | NABMI20 | AAEL009932 | lethal giant larva homologue | | AGAP004483 | Lethal(2) giant larvae protein. | 74 |
| 18 | NABNQ40 | AAEL003893 | DNA repair protein xp-c / rad4 | | AGAP012599 |  | 6974 |
| 18 | NABOE72 | AAEL006253 | conserved hypothetical protein | | AGAP001365 | Serine protease gd precursor | 8063 |
| 18 | NABS917 | AAEL005663 | centrin | | AGAP009260 | CG31802 | 5509 |
| 18 | NABW750 | AAEL007402 | peroxisome proliferator-activated receptor binding protein | | AGAP012219 |  |  |
| 18 | NABWD25 | AAEL007597 | serine protease | | AGAP004318 |  |  |
| 18 | NABWS14 | AAEL001425 | conserved hypothetical protein | | AGAP007402 | CG32584 | 5515 |
| 18 | NABY808 | AAEL001142 | rab gdp/GTP exchange factor | | AGAP002493 | Protein sprint | 5096 |
| 18 | NACBD45 | AAEL010135 | conserved hypothetical protein | | AGAP008875 | Putative conserved oligomeric Golgi complex component 4. | 15031 |
| 18 | NACMM64 | AAEL007470 | staufen | | AGAP007478 | Maternal effect protein staufen. | 3725 |
| 18 | NADXA28 | AAEL002387 | conserved hypothetical protein | | AGAP009811 | CDK5RAP3-like protein. | 79 |
| 19 | NAAFB68 | AAEL011455 | galactose-specific C-type lectin, putative | |  |  |  |
| 19 | NAAI221 | AAEL001959 | conserved hypothetical protein | | AGAP008036 | fledgling of Klp38B |  |
| 19 | NAAID42 | AAEL014863 | glycogenin | | AGAP007724 |  |  |
| 19 | NABM742 | AAEL011057 | DNA-repair protein complementing XP-A cells homolog | | AGAP007566 | DNA-repair protein complementing XP-A cells homolog | 6974 |
| 19 | NABNO19 | AAEL012998 | conserved hypothetical protein | | AGAP002707 | dpr18 | 7275 |
| 19 | NABOJ11 | AAEL004793 | dipeptidyl-peptidase | | AGAP000848 | CG9059 | 8236 |
| 19 | NABP074 | AAEL006196 | hemomucin | | AGAP004065 | Hemomucin | 9058 |
| 19 | NABPA74 | AAEL000476 | conserved hypothetical protein | | AGAP003225 | CG16969 | 5515 |
| 19 | NABPU76 | AAEL015533 | conserved hypothetical protein | | AGAP008558 | CG31326 | 4252 |
| 19 | NABQ656 | AAEL008730 | anillin/rhotekin (rtkn) | | AGAP010715 | Actin-binding protein anillin | 3779 |
| 19 | NABQD57 | AAEL006509 | hydrogen-transporting ATP synthase, G-subunit, putative | | AGAP009491 | lethal (2) 06225 | 15986 |
| 19 | NABWW76 | AAEL006859 | Myb-interacting protein, putative | | AGAP003410 | Myb-interacting protein 40 | 122 |
| 19 | NABXJ43 | AAEL007717 | alpha-endosulfine, putative | | AGAP010476 | endosulfine | 7584 |
| 19 | NABY746 | AAEL000508 | fibrinogen and fibronectin | | AGAP001554 |  |  |
| 19 | NACB120 | AAEL003508 | serine-pyruvate aminotransferase | | AGAP010387 |  |  |
| 19 | NACBN19 | AAEL014144 | rapsynoid | | AGAP002648 | CG6915 | 5488 |
| 19 | NACBQ43 | AAEL005422 | pyrroline-5-carboxylate dehydrogenase | | AGAP004366 | CG7145 | 6561 |
| 19 | NADCB50 | AAEL014261 | zinc finger protein | | AGAP003649 |  |  |
| 19 | NADCH67 | AAEL011447 | 60S ribosomal protein L14 | | AGAP005991 | 60S ribosomal protein L14. | 3735 |
| 19 | NADCK63 | AAEL009596 | sterol o-acyltransferase | | AGAP012216 | CG8112 |  |
| 19 | NADD507 | AAEL014583 | 60S acidic ribosomal protein P2 | | AGAP003025 | 60S acidic ribosomal protein P2 | 3735 |
| 19 | NADDQ52 | AAEL014396 | protein farnesyltransferase alpha subunit | | AGAP011767 | CG2976 | 18346 |
| 19 | NADDS75 | AAEL001126 | rest corepressor (corest) protein | | AGAP002488 |  | 45449 |
| 19 | NADE829 | AAEL002550 | polyA-binding protein interacting protein, putative | | AGAP001639 | polyA-binding protein interacting protein 2 | 1558 |
