# Supplementary material for: Comparative Expression Profiles of Midgut Genes in Dengue Virus Refractory and Susceptible Aedes aegypti across Critical Period for Virus Infection
Source: PLoS One. 2012 Oct 15;7(10):e47350. doi: 10.1371/journal.pone.0047350 (PMC3471866; doi:10.1371/journal.pone.0047350)
Supplement: Table S6 — Number of significant genes related to different pathways (KEGG). The list of pathways with significant genes (at least three) identified from the array analysis are shown. The total number of genes annotated by KEGG to the corresponding pathway are also listed for comparison. (DOCX) [file pone.0047350.s009.docx]

| **Pathway** | **No. of significant genes** | **Total no. of annotated genes of the pathway** |
| --- | --- | --- |
| mRNA surveillance pathway | 15 | 76 |
| Ribosome | 14 | 75 |
| Endocytosis | 13 | 79 |
| Oxidative phosphorylation | 13 | 95 |
| Purine metabolism | 13 | 106 |
| Protein processing in endoplasmic reticulum | 11 | 112 |
| Glycerophospholipid metabolism | 10 | 42 |
| Glycolysis / Gluconeogenesis | 10 | 31 |
| N-Glycan biosynthesis | 9 | 38 |
| RNA degradation | 9 | 57 |
| RNA transport | 9 | 133 |
| Ubiquitin mediated proteolysis | 9 | 88 |
| Butanoate metabolism | 8 | 18 |
| Pentose phosphate pathway | 8 | 16 |
| Peroxisome | 8 | 63 |
| Nucleotide excision repair | 7 | 40 |
| Proteasome | 7 | 39 |
| Wnt signaling pathway | 7 | 67 |
| ABC transporters | 6 | 14 |
| ECM-receptor interaction | 6 | 13 |
| MAPK signaling pathway - fly | 6 | 20 |
| Phagosome | 6 | 51 |
| Porphyrin and chlorophyll metabolism | 6 | 20 |
| Regulation of autophagy | 6 | 12 |
| Valine, leucine and isoleucine degradation | 6 | 34 |
| Amino sugar and nucleotide sugar metabolism | 5 | 45 |
| Ether lipid metabolism | 5 | 12 |
| Glutathione metabolism | 5 | 39 |
| Glycine, serine and threonine metabolism | 5 | 25 |
| Glycosaminoglycan degradation | 5 | 14 |
| Glycosphingolipid biosynthesis - globo series | 5 | 9 |
| Lysine degradation | 5 | 29 |
| mTOR signaling pathway | 5 | 27 |
| Pentose and glucuronate interconversions | 5 | 14 |
| Progesterone-mediated oocyte maturation | 5 | 49 |
| Riboflavin metabolism | 5 | 6 |
| Selenocompound metabolism | 5 | 10 |
| SNARE interactions in vesicular transport | 5 | 17 |
| Sphingolipid metabolism | 5 | 26 |
| Aminoacyl-tRNA biosynthesis | 4 | 20 |
| Arginine and proline metabolism | 4 | 38 |
| Basal transcription factors | 4 | 40 |
| Circadian rhythm - fly | 4 | 8 |
| Cysteine and methionine metabolism | 4 | 20 |
| DNA replication | 4 | 34 |
| Fanconi anemia pathway | 4 | 35 |
| Fatty acid biosynthesis | 4 | 8 |
| Fatty acid metabolism | 4 | 29 |
| Galactose metabolism | 4 | 23 |
| Hedgehog signaling pathway | 4 | 27 |
| Jak-STAT signaling pathway | 4 | 17 |
| minoacyl-tRNA biosynthesis | 4 | 22 |
| Neuroactive ligand-receptor interaction | 4 | 38 |
| Nicotinate and nicotinamide metabolism | 4 | 10 |
| Other types of O-glycan biosynthesis | 4 | 14 |
| Phosphatidylinositol signaling system | 4 | 35 |
| Protein export | 4 | 22 |
