# Supplementary material for: Comparative Expression Profiles of Midgut Genes in Dengue Virus Refractory and Susceptible Aedes aegypti across Critical Period for Virus Infection
Source: PLoS One. 2012 Oct 15;7(10):e47350. doi: 10.1371/journal.pone.0047350 (PMC3471866; doi:10.1371/journal.pone.0047350)
Supplement: Table S7 — List of primers that were used to perform qRT-PCR assays. The sequences of forward and reverse primer for each of the cDNAs investigated are listed in 5′–3′ direction. (DOCX) [file pone.0047350.s010.docx]

Table S7. List of primers those were used to perform qRT PCR assays.

| cDNA | Forward primer | Reverse primer |
| --- | --- | --- |
| NAAI041 | ATTTTGCCCGCGAATCC | GGTAGCTGGCAGGAGTTTACTGA |
| NABNY43 | ACGATCCTAGGCAGAATAAATGGT | CACAGCCCAGATGCTTTCG |
| NABOA44 | ATTAAACGCGTCCTCCAAAATC | GCGCGACGTGTTCACAGA |
| NABPJ55 | GCTACTGGCCGAAGGCAAA | TCCTTCAGGGCCCAGTTG |
| NABWN57 | CGCTGCCACCTACGAAGTC | CACCGTTCGAGCCAAGCTT |
| NABZ773 | TCAGGTCGGCCAGAACCA | TATCGGAAGACGCGTTGTTG |
| NADDS62 | GAGGTGCTGAACGCAATGAA | TTCACGAACCGCCTGGAT |
| NADEF37 | CCGTCGACAAGGAGGAGTTG | TGTGCCATGTCGTCATATCGT |
